# Proton-selective conductance and gating of the lysosomal cation channel TMEM175

**DOI:** 10.1073/pnas.2503909123

**Published:** 2026-01-14

**Authors:** Tobias Schulze, Timon Sprave, Carolin Groebe, Jan Hendrik Krumbach, Magnus Behringer, Andre Bazzone, Rocco Zerlotti, Niels Fertig, Mike Althaus, Kay Hamacher, Gerhard Thiel, Christian Grimm, Oliver Rauh

**Affiliations:** ^a^Department of Biology, Membrane Biophysics, Technical University of Darmstadt, Darmstadt 64287, Germany; ^b^Walther Straub Institute of Pharmacology and Toxicology, Endolysosomal Ion Channel Research, Faculty of Medicine, Ludwig-Maximilians-Universität, Munich 80336, Germany; ^c^Institute of Pathophysiology, Physiology and Pathophysiology of Cortical Neuronal Networks, Biomedical Research Centre, University Medical Center of the Johannes Gutenberg University, Mainz 55128, Germany; ^d^Department of Biology, Computational Biology & Simulation, Technical University of Darmstadt, Darmstadt 64287, Germany; ^e^Nanion Technologies, Munich 80339, Germany; ^f^Institute for Functional Gene Analytics, Department of Natural Sciences, Bonn-Rhein- Sieg University of Applied Sciences, Rheinbach 53359, Germany; ^g^Department of Biosciences, University of Milan, Milan 20133, Italy; ^h^Department of Pharmacology/Immunology, Infection and Pandemic Research, Fraunhofer Institute for Translational Medicine and Pharmacology, Munich 80799, Germany; ^i^Department of Pharmacology, Faculty of Medicine, University of Oxford, Oxford OX1 3QT, United Kingdom

**Keywords:** TMEM175, proton channel, patch-clamp, MD simulations, SSME

## Abstract

The lysosomal cation channel TMEM175 plays a key role in luminal pH homeostasis and lysosome function, with aberrant activity linked to Parkinson’s disease. Although initially described as a K^+^-selective channel, TMEM175 exhibits substantial H^+^ permeability. Here, we dissect complex changes affecting human TMEM175 conductance and ionic properties of TMEM175-mediated current in response to pH shifts on the luminal side of the protein. A drop in pH from 7.4 to 4.7 on the side equivalent to the lysosomal lumen triggers a sustained increase in TMEM175-mediated inward and outward currents, which is accompanied by a transient shift in the reversal potential (E_rev_) toward the theoretical equilibrium voltage for H^+^, yet remaining ~100 mV below the expected value even in the absence of K^+^. This discrepancy, along with low sensitivity of E_rev_ to the concentration gradient for K^+^, supports a model in which TMEM175-mediated H^+^ flux rapidly collapses the lysosomal pH-gradient. Molecular dynamics simulations identify H57 as a key residue on the luminal side of the open channel, which forms intra- and intersubunit salt bridges with D279 and E282. Supporting the functional importance of these interactions, the TMEM175 mutant H57Y displayed reduced H^+^- and K^+^-conductance and a reduced H^+^/K^+^ selectivity in whole-cell and lysosomal electrophysiological analyses. Our findings contribute to a better understanding of TMEM175’s complex electrophysiological properties, thereby expanding the possibilities of understanding the channel’s function in lysosomal physiology and pathophysiology.

## Significance

Malfunction of the lysosomal ion channel TMEM175 disrupts luminal pH homeostasis and has been linked to neurodegenerative disorders, such as Parkinson’s disease. The channel’s principal ion selectivity remains a subject of ongoing debate, with conflicting evidence supporting K^+^ or H^+^ as the dominant permeant species. To investigate the channel’s selectivity and pH dependence, we analyzed TMEM175-mediated currents in response to changes in luminal pH. Electrophysiological recordings revealed that luminal acidification activates a H^+^-conductance, leading to rapid collapse of the pH-gradient. Integrating experimental and computational approaches, we identified H57 as a key residue regulating TMEM175-mediated H^+^ flux. Presented findings deepen our understanding of human TMEM175 structure–function and broaden the possibilities for developing therapeutic approaches for the treatment of TMEM175-associated neurodegenerative diseases.

Transmembrane protein 175 (TMEM175) is a ubiquitously expressed cation channel localizing to endosomal and lysosomal membranes. Its dysfunction has been identified as a key risk factor for neurodegenerative disorders, including Parkinson’s disease (1). TMEM175 was initially described as a lysosomal K^+^ channel (2), proposed to maintain luminal pH homeostasis and lysosome function (2, 3). While follow-up studies confirmed a dominant K^+^-conductance at neutral pH, it was shown that acidic pH on the luminal side (pH_lum_) of TMEM175 strongly increases channel conductance and shifts ion selectivity in favor of H^+^ (4, 5). Complementary patch-clamp studies demonstrated that the reversal potential of TMEM175-mediated currents (E_rev_) shifts as a function of the transmembrane pH-gradient (ΔpH) in acidic pH_lum_ buffers. While these observations are consistent with a selective H^+^-conductance, absolute E_rev_ values remain far below the theoretical Nernst potential for protons (E_H+_) even when N-methyl-D-glucamine (NMDG^+^) was the only other cation available for transport (4, 5). Accordingly, reported E_rev_ values do not match with those expected for a purely H^+^-selective channel. A scenario where TMEM175 not only transports H^+^, but to some extent larger ions, even including the large impermeable cation NMDG^+^, could explain this discrepancy (4, 5), however, the narrow geometry of the TMEM175 channel pore conflicts with this interpretation (6, 7).

A more plausible scenario is that measured E_rev_ values deviate from the calculated E_H+_ due to experimental limitations. Establishing the correct Nernst potential for H^+^-conducting channels is a well-known problem in electrophysiological experiments (8–10). The low concentration of H^+^ in solution, along with rapid diffusion of H^+^ relative to slowly moving buffer molecules, make it nearly impossible to precisely control H^+^ concentrations at entrance and exit of H^+^-conducting channel pores (10). This problem is particularly pronounced in channels that generate high H^+^-mediated currents, as this induces an unavoidable dissipation of pH-gradients (8–10). The resulting drift in E_rev_ cannot be prevented, even with high pH buffer concentrations. A similar dynamic shift in E_rev_ is also elicited by the H^+^ uncoupler carbonyl cyanide-*p*-trifluoromethoxyphenylhydrazone (FCCP) (9), suggesting it is a general feature of H^+^-conducting molecules. Hence, we expect E_H+_ to change over time in response to any proton-conductance, including that mediated by TMEM175. Since the biophysical mechanisms of H^+^ permeation and gating of this ion channel remain poorly understood, we evaluated the relationship between TMEM175-mediated H^+^-conductance and changes in E_rev_. We show that acidification on the luminal side of TMEM175 induces a transient positive shift in E_rev_, indicating predominant H^+^-conductance. This is followed by a conductance-dependent return of E_rev_ to values near 0 mV, which is due to gradual dissipation of the proton gradient across the membrane. Using whole-cell and lysosomal patch-clamp experiments as well as solid-supported membrane electrophysiology (SSME) on isolated lysosomes, we provide evidence for dual K^+^- and H^+^-permeability in TMEM175. Guided by TMEM175 structures resolved in open and closed conformations (7), we also identified histidine H57 as a pH-sensor on the luminal side of the protein which modulates ion conductance, H^+^/K^+^ selectivity, and channel gating.

## Results and Discussion

To characterize the pH-induced conductance in TMEM175, the protein was transiently overexpressed in HEK293 cells. This leads to plasma membrane insertion of functional TMEM175 channels, adopting an orientation in which the luminal-facing side of the protein is exposed to the extracellular solution (6). We therefore used patch-clamp recordings in the whole-cell configuration to monitor TMEM175-mediated currents in response to changes in the extracellular (=luminal) solution. Currents were first measured in symmetrical buffers containing 140 mM potassium methanesulfonate (KCH_3_SO_3_; K-MS), 10 mM TEA-OH, and 5 mM Cs-MS at pH 7.4. The large impermeable MS anion was used to suppress endogenous pH-sensitive anion channels. Cs^+^ and TEA^+^ were included to block most of the endogenous K^+^ channels without affecting TMEM175 conductance (2, 4).

**Fig. 1.**
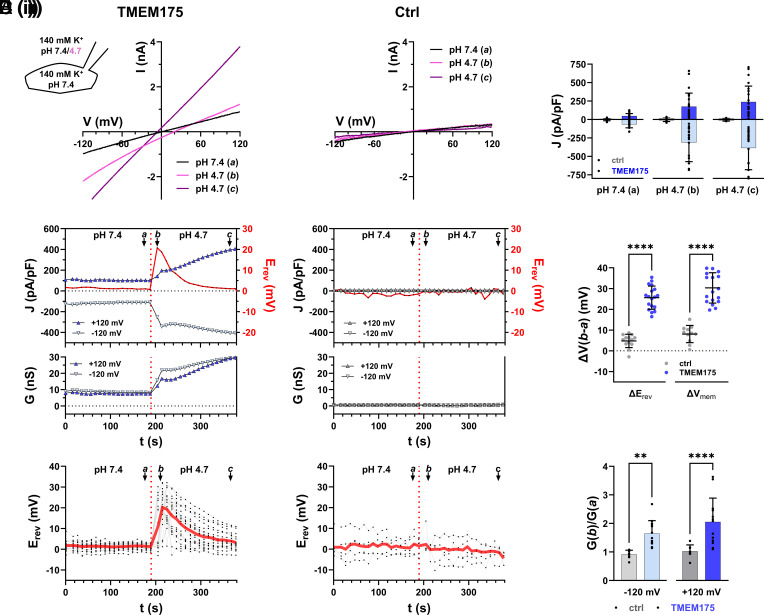
Acidification of the luminal side induces a continuous increase in conductance but only a transient shift of E_rev_ to positive values in TMEM175-expressing cells. (*A* and *B*) Whole-cell recordings of TMEM175-expressing (*A*) and GFP-expressing (empty vector transfected) control cells (Ctrl) (*B*). Bath and pipette solution contained 140 mM K-MS. The internal pH (pH_in_) was 7.4. The external pH (pH_ex_) was changed from 7.4 to 4.7. (*i*) Representative current responses to voltage-ramps from +120 to −120 mV shortly before (*a*, black), shortly after (*b*, pink), and 3 min after (*c*, purple) a pH_ex_-jump from 7.4 to 4.7 (see also panels (*ii*) for time indices *a–c*). (*ii*) Corresponding time-courses of reversal voltage (E_rev_) as well as current densities (upper graph) and chord conductance (lower graph) at ±120 mV of TMEM175-expressing and control cells. Values from voltage-ramp recordings as in (*i*). (*iii*) Individual (black) and averaged time-courses (red) of E_rev_ from pH_ex_ jump experiments as shown in (*ii*). (*C*) Current densities at ±120 mV at time points *a*, *b,* and *c* in (*A*) and (*B*). (*D*) Maximal change in E_rev_ and free running membrane voltage V_mem_ in response to pH_ex_-jump from 7.4 to 4.7 of control (gray) and TMEM175-expressing (blue) cells. (*E*) pH_ex_-jump-induced change in chord conductance at ±120 mV for control (gray) and TMEM175-expressing (blue) cells. Bars in (*C*–*E*) represent arithmetic mean ± SD; values from individual recordings are shown as closed circles. Statistical analyses in (*D*) and (*E*) were performed with unpaired two-way ANOVA with Sidák’s and Tukey’s multiple comparison test, respectively.

Fig. 1 shows the electrical parameters of cells expressing TMEM175 (Fig. 1*A*) or GFP (empty vector transfected control cells) (Fig. 1*B*) each subject to voltage-ramps (±120 mV; 0.46 mV/ms, *SI Appendix*, Fig. S1*A*) repeated every 10 s. This allowed continuous monitoring of changes in current/voltage (I/V) relationships, current densities (J), membrane conductance (G) at ±120 mV and E_rev_ (Fig. 1 *A*–*E*). At neutral external solution pH (pH_ex_), both TMEM175-expressing and control cells exhibited small currents with E_rev_ values around 0 mV (Fig. 1 *A*, *iii* and *B*, *iii*). Inward and outward current densities (J) were higher in TMEM175-expressing cells than in control cells (Fig. 1*C*), consistent with a K^+^-conductance of the TMEM175 channel under this condition.

Acidification of pH_ex_ from 7.4 to 4.7 elicited a continuous increase of current densities and conductance in TMEM175-expressing cells (n = 26) (Fig. 1*A*). This was accompanied by a shift in E_rev_ from 0 mV to a peak near +20 mV before returning back toward baseline (Fig. 1 *A, iii* and *C–E*). By contrast the GFP-expressing control along with n = 11 additional cells, exhibited no detectable change in E_rev_, current densities, or conductance (Fig. 1 *B*, *C*, and *E*) in response to the same pH change. Consequently, acid-induced increase in conductance and transient excursion in E_rev_ observed in TMEM175-expressing cells can be directly attributed to the activity of the TMEM175 channel. Notably, the acid-induced transient shift of E_rev_ in TMEM175-expressing cells has not been reported in previous studies on TMEM175 (4, 5).

Studies on other H^+^-conducting channels reported that, under voltage-clamp conditions, their activity unavoidably causes local depletion and/or accumulation of H^+^ at entry and exit of the channel protein (8, 10). To minimize any potential directional H^+^ accumulation in response to voltage-ramps, we applied them in forward and reverse directions (from +120 to −120 mV and back) (*SI Appendix*, Fig. S1). Scrutiny of E_rev_ values shows that the E_rev_ of the reverse ramp is left shifted by ~5 mV compared to the E_rev_ of the forward ramp (*SI Appendix*, Fig. S1 *B* and *C*), indicating that the ramp-evoked H^+^ inward current indeed caused some erosion of the pH-gradient. To correct for this minor ramp-induced artifact all reported E_rev_ values represent the average of forward and reverse ramps measurements.

To fully eliminate any impact of the clamp voltages on E_rev_, we recorded the free-running membrane voltage (V_mem_) in TMEM175-expressing cells before and after a pH_ex_ jump from 7.4 to 4.7 in the current-clamp mode. This procedure also triggered a transient depolarization of V_mem_ in response to the pH jump (Fig. 1*D* and *SI Appendix*, Fig. S2). Assuming that V_mem_ is dominated by E_rev_ of TMEM175 in this setting, our data confirm that transient excursions of E_rev_ are not an artifact of the recording methodology, but a consequence of TMEM175 ion channel activity.

Having excluded experimental artifacts as the source of transient E_rev_ shifts observed in initial experiments, three plausible explanations remain for their occurrence: 1) an acid-induced increase in TMEM175 H^+^-conductance may only be short-lived; 2) the pH_ex_-jump may elicit a rapid H^+^- and a delayed dominating K^+^-conductance of TMEM175; and 3) the TMEM175 H^+^-conductance may cause a time-dependent dissipation of the pH-gradient across the membrane.

A transient shift of E_rev_ because of a short-lived increase in H^+^-conductance is not supported by the data. Notably, the acid-induced inward and outward conductance continuously increases while E_rev_ already shifts back toward 0 mV (Fig. 1 *A, ii*).

To test if a combination of a rapid rise in H^+^-conductance and a delayed increase in K^+^-conductance can account for the transient shift in E_rev_, we modified the experiments shown in Fig. 1*A*: once E_rev_ had settled close to 0 mV after the pH_ex_-jump, the external K^+^ concentration [K^+^]_ex_ was reduced 10-fold (140 to 14 mM) (*SI Appendix*, Fig. S3*A*). If the backshift of E_rev_ originated from a delayed and dominating K^+^-conductance, E_rev_ should settle close to the K^+^ Nernst potential (E_K+_) of −60 mV in [K^+^]_ex_ = 14 mM. The 10-fold reduction in [K^+^]_ex_ had in control cells no appreciable effect on the membrane conductance (*SI Appendix*, Fig. S3*C*) and their E_rev_ dropped by −4 ± 1 mV (n = 6) (*SI Appendix*, Fig. S3*B*). By contrast, the same 10-fold reduction in [K^+^]_ex_ shifted E_rev_ by −15 ± 4 mV (n = 5) in TMEM175-expressing cells (*SI Appendix*, Fig. S3*B*). Notably, the latter remains significantly more negative than in control cells, but still well positive of the expected E_K+_ of −60 mV. Hence, TMEM175 conductance seems not to be dominated by K^+^ in acidic pH_ex_, when the latter is strongly buffered. This relationship may change in experimental conditions in which pH_ex_ is only very weakly buffered (11).

To further examine a contribution, if any, of K^+^ to the transient shift in E_rev_, we repeated experiments as shown in Fig. 1 employing solutions in which external and internal K^+^ was replaced by NMDG^+^ (*SI Appendix*, Fig. S3 *D*–*G*). In the absence of K^+^, TMEM175-expressing cells showed very low current densities (*SI Appendix*, Fig. S3 *D* and *E*) at pH_ex_ = 7.4, which were not appreciably different from control cells at ±120 mV (*SI Appendix*, Fig. S3*E*). In TMEM175-expressing cells, changing pH_ex_ from 7.4 to 4.7 triggered the same shifts in conductance and E_rev_ as observed in the presence of K^+^: the pH_ex_-jump caused a continuous increase in currents and a transient positive excursion of E_rev_ (*SI Appendix*, Fig. S3 *D*–*G*). This was not the case in control cells. These data strongly suggest that the acid-induced shifts in E_rev_ are an unavoidable consequence of the H^+^-conductance and that the acid-induced H^+^-currents are mechanistically independent of K^+^-currents. In the absence of K^+^, TMEM175 conductance is entirely dominated by H^+^, while it is determined by a mixture of K^+^- and H^+^- conductance in the presence of K^+^.

**Fig. 2.**
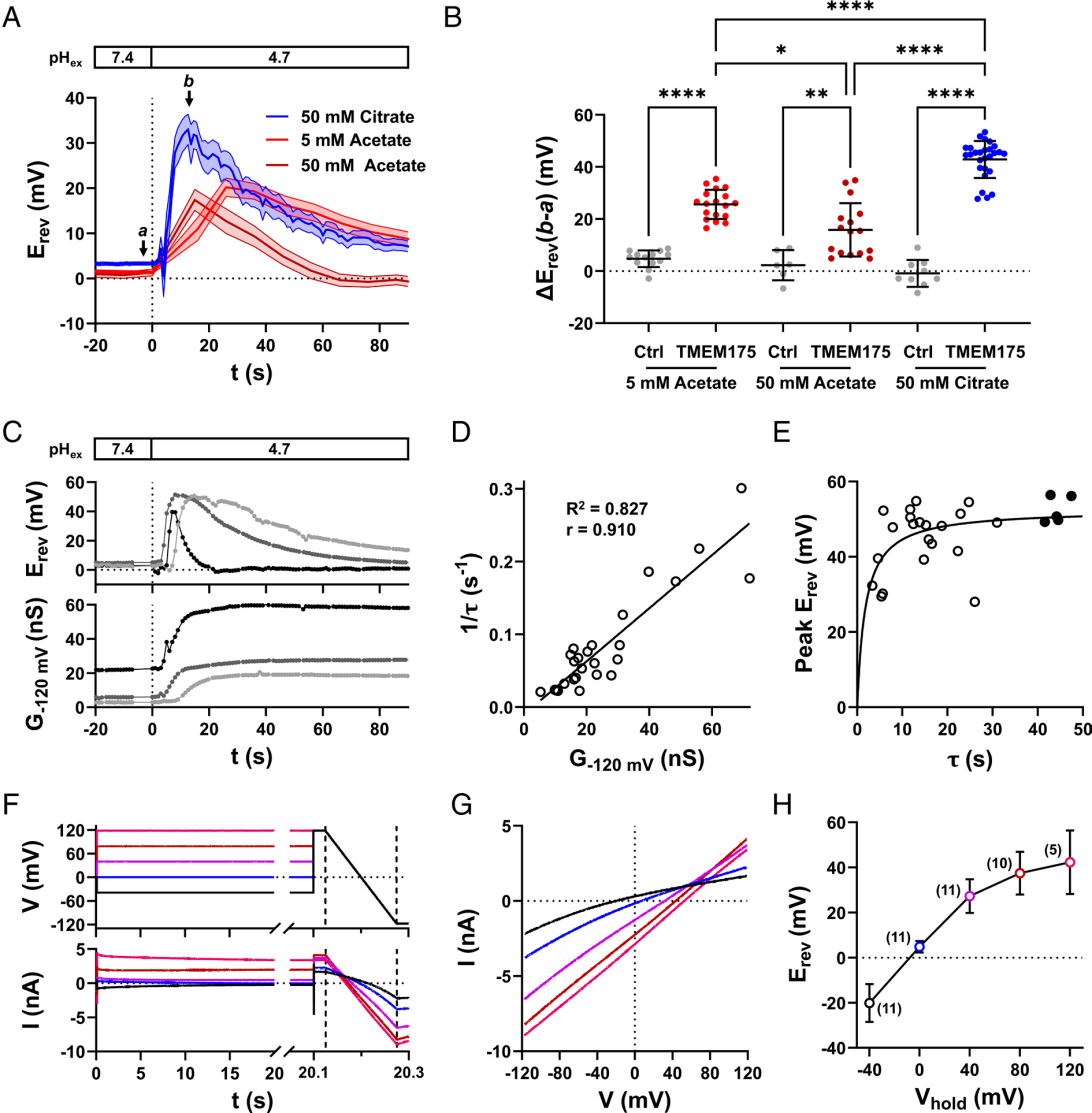
The TMEM175-mediated H^+^-current causes a time-dependent erosion of the pH-gradient in whole-cell patch-clamp recordings. (*A*) Averaged time-courses ± SEM of E_rev_ of TMEM175-expressing cells in symmetrical 140 mM K^+^ concentrations, in response to a pH_ex_ jump from 7.4 to 4.7 under different pH-buffering conditions. The internal and external solutions were buffered to pH 7.4 with 5 mM (light red) or 50 mM HEPES (dark red and blue). The external solution was buffered to pH 4.7 with 5 mM acetate (light red), 50 mM acetate (dark red), or 50 mM citrate (blue). (*B*) Maximal change in E_rev_ in response to pH_ex_ jumps from 7.4 to 4.7 in control (ctrl, gray) and TMEM175-expressing cells with different pH 4.7 buffer conditions as shown in panel *A*. Statistical comparisons were done by unpaired two-way ANOVA with Tukey’s multiple comparison test. (*C*) Time-courses of E_rev_ and corresponding chord conductance at −120 mV (G_−120 mV_) of three TMEM175-expressing cells in response to pH_ex_ jumps from 7.4 (50 mM HEPES) to 4.7 (50 mM citrate). (*D*) Relationship between G_−120 mV_ (90 s after external acidification) and the reciprocal of the time constant (τ) of the E_rev_ backshift. The time constant was obtained by fitting the falling phase (backshift) of E_rev_ with a single exponential function. The straight line represents the best fit with a linear function. R^2^ and the Pearson correlation coefficient (r) are displayed in the graph. (*E*) Relationship between time constant of the E_rev_ backshift and the maximal E_rev_ value (Peak) measured upon acidification. The straight line represents the best fit with the Hill equation. Closed black circles highlight data points with τ > 40 s. These data points are further used for calculation of P_H+_/P_K+_ in Fig. 4. (*F*) Voltage pulse protocol (upper graph) and corresponding current response of a representative TMEM175-expressing cell, 90 s after acidification of the external solution. Internal and external solutions contained 140 mM K^+^ and were buffered with 50 mM HEPES to pH 7.4 and 50 mM citrate to pH 4.7, respectively. (*G*) Magnification of ramp currents shown in panel (*F*), plotted against the corresponding membrane voltage. Colors in (*F*) and (*G*) correspond to different holding potentials of voltage pulses shown in panel (*F*). (*H*) Arithmetic mean ± SD of E_rev_ values from (n) independent experiments as shown in (*G*), plotted as a function of V_hold_.

Taken together, the data are compatible with a scenario in which a jump from neutral to acidic pH augments a H^+^-current through TMEM175. The resulting H^+^ inward current generates a gradual and eventually complete dissipation of the pH-gradient. Such local variations in proton concentration, that were also observed in other H^+^ channels, could be reduced, but not fully abolished, by high pH buffer concentrations (9, 10, 12–14). To examine the contribution of pH buffering on the acid-induced transient E_rev_-shift in TMEM175-expressing cells, experiments as shown in Fig. 1 were repeated using buffers at concentrations 10-fold higher than those used initially (50 mM HEPES for pH 7.4 and 50 mM acetate for pH 4.7) (Fig. 2). Since acetate is membrane-permeable in its protonated form, we also substituted acetate with the membrane-impermeable pH buffer citrate. We hypothesized that an elevated pH-buffering capacity may not abolish the transient shift of E_rev_, but slow down the erosion of the pH-gradient and in turn increase the E_rev_ peak value. While the electrical properties of control cells again did not change in response to the pH_ex_ jump from 7.4 to 4.7 under these conditions, the transient shift of E_rev_ to positive voltages was maintained in TMEM175-expressing cells (Fig. 2*A*). The acid-induced peak of E_rev_ was dependent on the pH buffers: In the presence of 50 mM citrate, E_rev_ peaked at +42.9 ± 6.9 mV (n = 27), which was significantly more positive than in 5 mM acetate (+25.6 ± 5.4 mV; n = 18) (Fig. 2 *A* and *B*). Interestingly, when 50 mM acetate was employed, E_rev_ was smaller (+15.9 ± 9.9 mV, n = 16), and the backshift toward 0 mV faster, compared to low buffer concentrations (Fig. 2 *A* and *B*). This at first glance counterintuitive result can be explained by the membrane-permeable acetate buffer, which can contribute to the collapse of the pH-gradient via a weak acid loading effect (15).

The data derived from experiments with 50 mM citrate buffer show that an elevated buffering capacity attenuates but does not abolish the erosion of the pH-gradient. Since the latter is a consequence of the TMEM175 mediated H^+^-current, we hypothesize that the kinetics of E_rev_ backshift is a function of the TMEM175 conductance. We therefore quantified the acid-induced increase in conductance and the corresponding exponential decay of the E_rev_ value back toward 0 mV in individual cells after switching the pH from 7.4 to either 4.7 or only 6.1.

Fig. 2*C* shows the responses of membrane conductance and E_rev_ of three exemplary TMEM175-expressing cells to acidification of the external solution to pH 4.7, in the presence of 50 mM citrate. Typically, cells with a larger increase in TMEM175 conductance showed a faster decay of E_rev_. The reciprocal of the time constant of the exponential E_rev_ decay (1/τ) rises linearly with increasing membrane conductance (Fig. 2*D*). A plot of the E_rev_ peak as a function of τ (Fig. 2*E*) gives a saturating curve suggesting an E_rev_ between +50 mV and +60 mV as maximal value under this condition.

When the pH was decreased from 7.4 to 6.1, only TMEM175-expressing cells showed a progressive increase in conductance together with a transient depolarization of E_rev_ by +9.2 ± 2.1 mV (n = 17) (*SI Appendix*, Fig. S4 *A*–*C*). The fact that this pH-induced increase in conductance could be abolished by 4-AP (*SI Appendix*, Fig. S4 *A* and *D*), a known inhibitor of TMEM175 channels (6, 7) underscores that already a mild acidification can cause an increase in TMEM175 conductance. In good agreement with the expectation that a smaller driving force for the proton current should generate a slower erosion of the pH gradient we observed that E_rev_ values decreased in pH 6.1 only slowly to +6.0 ± 3.1 mV over 90 s after acidification (*SI Appendix*, Fig. S4 *A* and *C*).

If the observed collapse of the pH-gradient is a function of the TMEM175 H^+^-conductance, we expect that the steady-state E_rev_ depends on the conditioning voltage to which cells are clamped prior to a ramp protocol (9, 16). To test this prediction, 90 s after acidification of the external solution to either pH_ex_ 4.7 or 6.1, TMEM175-expressing cells were clamped for 20 s to holding potentials between −40 and +120 mV. Afterwards, fast voltage-ramps were applied (Fig. 2 *F*–*H*). Data in Fig. 2*H* show that in pH_ex_ 4.7, the closer the conditioning voltage was to the theoretical H^+^ Nernst potential, the more positive was E_rev_. In other words, the lower the driving force for H^+^ influx and hence erosion of the pH-gradient prior to the ramp, the closer the measured E_rev_ approaches E_H+_. As predicted, this dependency turned out to be shallower under less acidic conditions. In pH_ex_ 6.1, the measured E_rev_ value is still sensitive to the conditioning voltage (*SI Appendix*, Fig. S4 *E* and *F*), albeit less than in pH_ex_ 4.7. In pH 7.4, this dependency is no longer apparent (*SI Appendix*, Fig. S4 *E* and *F*).

In summary, these data confirm that TMEM175 primarily conducts H^+^ after acidification of the external, i.e., luminal side, and that this proton-conductance causes a collapse of the pH-gradient in whole-cell patch-clamp recordings even at high pH-buffering capacity.

### H57 Plays a Key Role in Sensing the External/Luminal pH.

**Fig. 3.**
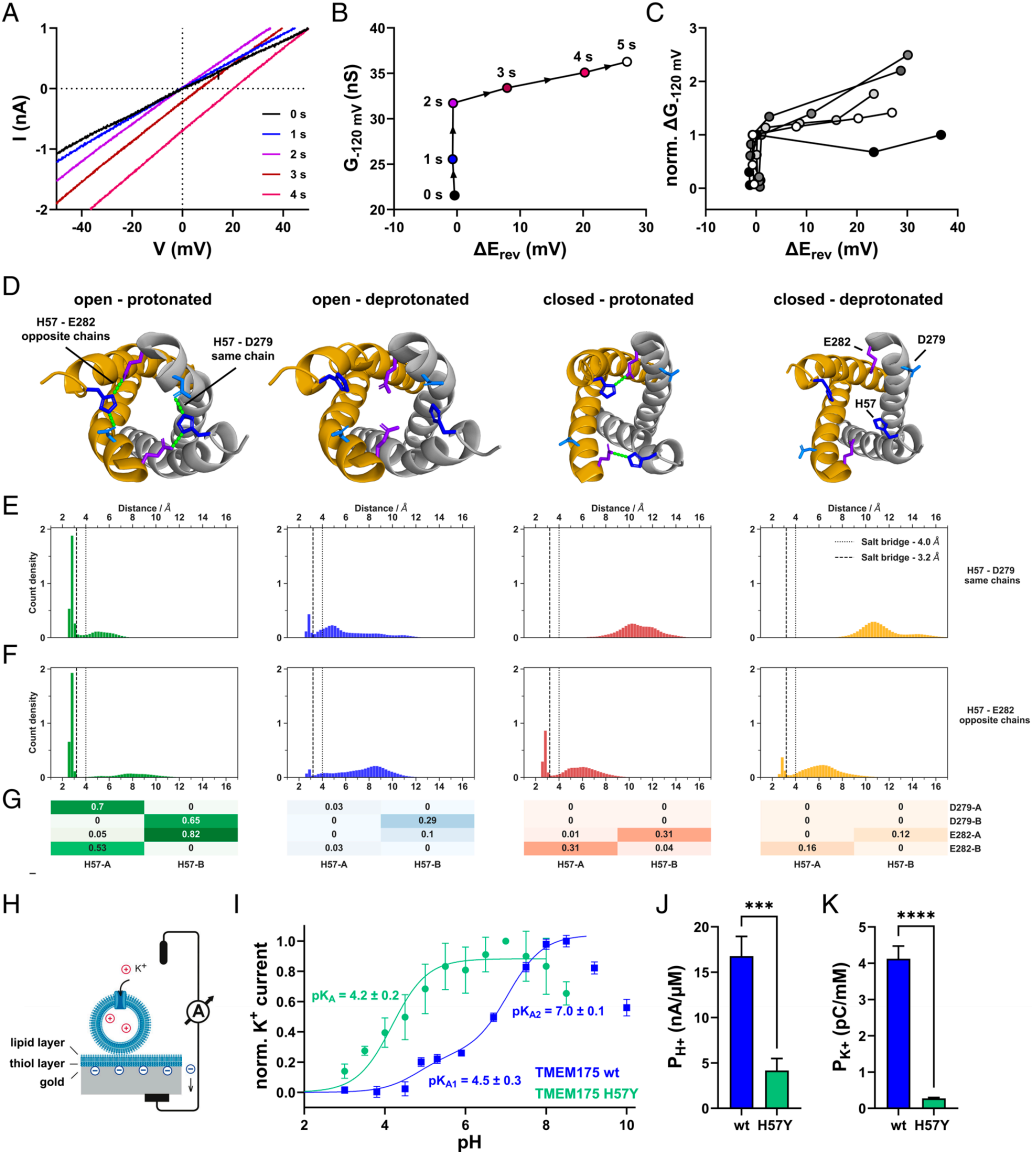
Histidine 57 is involved in sensing luminal acidification and gating of TMEM175. (*A*) Representative current responses of a TMEM175-expressing cell to voltage-ramps applied at an interval of 1 s immediately after a pH_ex_ jump from 7.4 (50 mM HEPES) to 4.7 (50 mM citrate), in symmetrical 140 mM K^+^ concentrations. The pH of the internal solution was 7.4. (*B*) Conductance at −120 mV calculated from ramps shown in (*A*), plotted against the corresponding change in E_rev_ (relative to the last voltage-ramp before the pH_ex_ jump). (*C*) Similar data as shown in (*B*), derived from five TMEM175-expressing cells. To enhance comparability, ΔE_rev_ values were plotted against normalized changes in conductance. The G_−120 mV_ value measured in response to the voltage-ramp before the pH_ex_-jump was therefore set to zero and the ΔG_−120 mV_ value measured directly before the onset of the E_rev_ shift to one. (*D*–*G*) Molecular dynamics (MD) simulations of wildtype (wt) TMEM175. (*D*) Snapshots of TMEM175 in the open and closed state with H57 either protonated or deprotonated. Intramonomer salt-bridges between H57 and D279 as well as the intermonomer interactions between H57 and E282 are displayed as green lines. (*E* and *F*) Aggregated distances between H57-D279 (*E*) and H57-E282 (*F*) during simulations in these states are depicted as histograms, with reference salt-bridge distances indicated by dotted (4.0 Å) and dashed lines (3.2 Å). Each histogram is normalized such that its total area equals 1. (*G*) Heatmap summary of the luminal salt-bridge network as share of MD frames below a distance threshold of 3.2 Å for the corresponding channel-state group (shown in *F*), for all possible H57-D279/E282 distance pair combinations in both TMEM175 chains. Minimum distances between each of the two imidazole-nitrogen atoms in H57 and carboxy-oxygens in D279/E282 are drawn from four replica MD simulations for the start structure in the respective state, with a total simulation time of 3.5 µs per state. Prior to the analysis of aggregated distances, datapoints from the first 50 ns of each MD trajectory were discarded. All underlying distance timeseries for full-length trajectories are depicted in *SI Appendix*, Figs. S6 and S7. (*H*–*J*) SSME recordings on lysosomes isolated from TMEM175 wt- and H57Y-expressing HEK293 cells. (*H*) Schematic representation of an SSME experiment. (*I*) pH dependence of K^+^ flux assessed using 50 mM [K^+^]-jumps at varying pH. Data for TMEM175 wt were adapted from ref. 17. Data points represent arithmetic means ± SD from n = 5 (wt) and n = 8 (H57Y) independent sensors. (*J*) H^+^-permeabilities (P_H+_) for TMEM175 wt and TMEM175 H57Y from pH-jump experiments in *SI Appendix*, Fig. S8*B* in the absence of K^+^. (*K*) K^+^-permeabilities for TMEM175 wt and H57Y from K^+^ flux experiments in *SI Appendix*, Fig. S8*A*. External and intralysosomal pH was 7.6. P_K+_ values were calculated from K^+^ flux data between [K^+^] = 32 and 80 mM.

A combination of pH buffers at high concentration and a 10-fold higher frequency of ramp protocols allowed for a better temporal resolution of early events that are triggered by acidic pH_ex_. Recordings from an exemplary cell in Fig. 3*A* revealed that a pH_ex_-jump from 7.4 to 4.7 produces an immediate increase in conductance, while E_rev_ remains near 0 mV during initial ramps. Then, after a delay, a positive shift of E_rev_ occurs while the rise in conductance continues (Fig. 3 *A* and *B*). This temporal uncoupling between an acid-induced increase in conductance and shift in E_rev_ was observed in several cells (Fig. 3*C*) suggesting a dual effect of the acidic pH_ex_: First, TMEM175 K^+^-conductance present at neutral pH is enhanced, then channel selectivity is altered, allowing an additional H^+^-conductance. From this sequential pH-effect we expect that the same pH_ex_-jump in [K^+^]_ex_ = 14 mM should first shift E_rev_ negative toward E_K+_ at −60 mV before it shifts positive toward E_H+_. In fact, we observed such a shift of around 3 to 5 mV in such experiments (*SI Appendix*, Fig. S5).

In search for structural elements and mechanisms of such a pH dependency, we scanned the cryoEM-structures of the channel in its presumed open and closed state (7) for potential candidate sites that could mediate a pH-sensitivity in the range between 7.4 and 4.7 from the luminal side. Functional TMEM175 channels are homodimers, with subunits composed of two homologous but nonidentical repeats of six transmembrane α-helices. Given that regulation of gating by pH is likely driven by protonation–deprotonation events on the luminal side of the channel, we focused on histidine 57 (H57), a luminally exposed residue of previously identified functional significance (4). In the open structure, protonated H57 lies in close proximity to D279 on the same subunit and to E282 of the opposing, enabling formation of salt-bridge interactions between H57 and both residues (Fig. 3*D*). Such structural organization may keep the entrance to the channel pore open and might provide a pathway for H^+^- and K^+^-conductance. The potential gating function of H57 is further supported by the finding that the expected salt bridge partners are far apart from each other in the closed channel structure (Fig. 3*D*). Hence, protonation of H57 probably stabilizes the salt bridge network at the luminal entrance to the pore which may initiate gating to the open state.

To investigate this hypothesis, we performed MD simulations using the structure of TMEM175 in open and closed conformation. For each of the two structures, as well as for each H57 protonation state—i.e., protonated and deprotonated—four individual simulations were run (Fig. 3*D*). We monitored distances between H57 and candidate interaction partners for salt bridge formation: D279 (intrachain) and E282 (interchain) (Fig. 3 *E*–*G*). Only for the open-protonated state, the critical amino acids are most of the time below threshold distances required for salt bridge formation. By contrast, markedly higher distances as well as a wider distribution of distances were observed for the open-deprotonated state (Fig. 3 *E*–*G*). For both closed states, most distances between amino acid distance pairs are beyond the salt-bridge threshold.

Even for the open-protonated state, a relatively high variability between the two subunits was observed for H57-E282 distances. This can be seen in higher distances for one of the two distance pairs (Fig. 3*G*), as well as in prolonged time frames with larger distances between H57 in subunit A (H57-A) and E282 in subunit B (E282-B) (*SI Appendix*, Fig. S7).

Taken together, the MD simulation results are compatible with a pH-dependent salt bridge formation mediated by H57 in TMEM175. These salt bridges could stabilize the open pore while their disruption, after deprotonation of H57, might destabilize this configuration. However, the variability of the simulation data and the finding that salt bridge formation in the open-deprotonated channel state does not always show the same stability for both intermonomer contacts of H57-E282, suggests a more complex and dynamic system which is not exclusively relying on a H57-mediated salt bridge formation (Fig. 3*G* and *SI Appendix*, Fig. S7, last row).

Our whole-cell patch-clamp experiments suggest that TMEM175 conductance in lysosomes should depend on the luminal pH (pH_lum_). Further, MD simulations suggest involvement of protonated H57 in salt bridge formation within the upper luminal pore, consistent with the proposed role of H57 as a pH-sensor and mediator that maintains the open channel state.

Previous reports employing SSME recordings on lysosomes have shown that the K^+^-conductance of TMEM175 (Fig. 3 *H* and *I*) is pH-dependent, but with two distinct pK_A_ values (pK_A1_ = 4.5 ± 0.3 and pK_A2_ = 7.0 ± 0.1), with the latter being close to the expected pK_A_ value of a histidine side chain (17). To examine if pK_A2_ is linked to a protonation of H57, we substituted H57 by tyrosine (Y) and repeated the SSME experiments with lysosomes isolated from TMEM175 H57Y-expressing HEK293 cells. The H57Y mutant channel lacks the titratable H57 side chain but maintains the aromaticity and similar steric properties in this position. The respective titration curve of the K^+^-conductance, in which the pH was identical on both sides of the membrane, shows that wt and mutant channels conduct K^+^ at moderate alkaline pH and that this conductance is declining with acidic pH values (Fig. 3*I*). Most importantly, while the decay of conductance of the wt channel exhibits two pK_A_ values (17), the mutant shows only one. The finding that the pK_A_ of 4.2 is conserved in mutant and wt and that the pK_A_ of 7.0 is absent in the H57Y mutant, underscores the importance of H57 in the control of TMEM175 function. However, it is important to note that moderate acidic pH suppresses the K^+^-conductance in the wt channel but not in the mutant. The suppression of the K^+^-current can be interpreted as competition between H^+^ and K^+^ fluxes, suggesting that both cations share a common permeation pathway in the water-filled central pore, as has been postulated by others (5, 7). Consequently, the H57Y-induced change in the pH_ex_-dependent K^+^-conductance suggests that H57 plays a role in gating of the H^+^-conductance.

Additional SSME measurements show that the substitution H57Y not only reduces H^+^-permeability by a factor of about 4 (Fig. 3*J* and *SI Appendix*, Fig. S8*B*), but also causes a 15-fold reduction in lysosomal K^+^-permeability (Fig. 3*K* and *SI Appendix*, Fig. S8*A*), demonstrating that H57 plays a critical role for H^+^- and K^+^-permeation through TMEM175.

Whole-lysosome patch-clamp recordings support this conclusion, as lysosomes carrying the H57Y mutant show only slightly higher currents compared with control lysosomes at both pH_lum_ = 7.4 and pH_lum_ = 4.7 (*SI Appendix*, Fig. S9 *D*, *F*, and *G*). By contrast, lysosomes isolated from cells expressing TMEM175 wt exhibit much larger currents, which are sensitive to the known TMEM175 blocker 4-AP (*SI Appendix*, Fig. S9 *A*–*C*). On average, current densities of TMEM175-expressing lysosomes at +120 mV and −120 mV are 3.9-fold and 4.7-fold higher, respectively, relative to control lysosomes or lysosomes expressing the H57Y mutant channel (*SI Appendix*, Fig. S9 *F* and *G*). V_mem_ of TMEM175-expressing organelles is depolarized to +31.8 ± 4.0 mV (n = 6) after acidification of the lysosomal lumen (*SI Appendix*, Fig. S9*E*) while the same acidification of the lumen of control lysosomes (n = 6) and lysosomes carrying the H57Y mutant (n = 6), has no measurable effect on V_mem_, which remained close to 0 mV in both cases (*SI Appendix*, Fig. S9*E*).

**Fig. 4.**
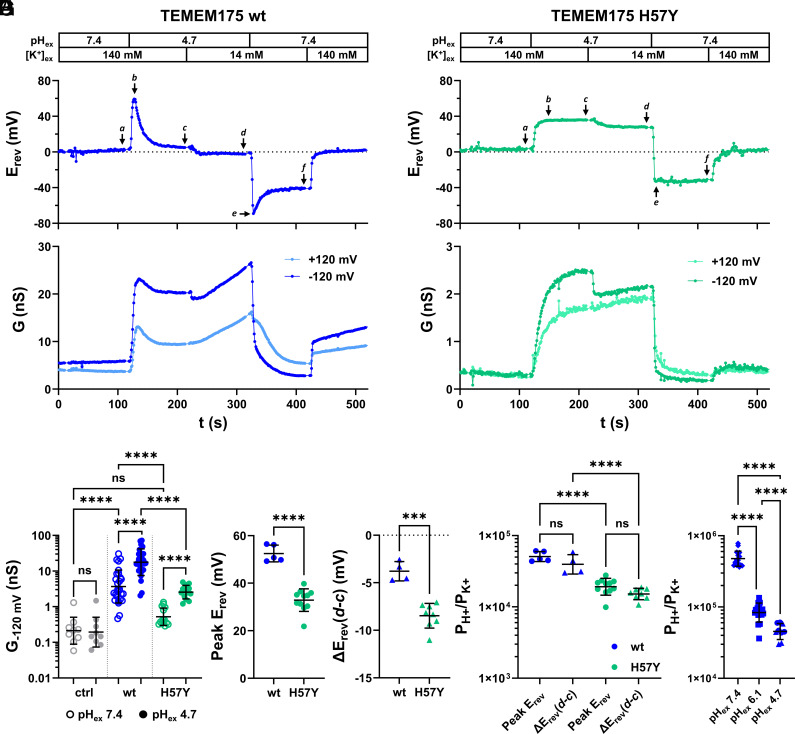
Substitution of H57 with the nonprotonatable amino acid tyrosine alters conductance and H^+^/K^+^ selectivity of TMEM175 at low pH_ex_. (*A* and *B*) Time-courses of E_rev_ (upper graph) and chord conductance at ±120 mV (lower graph) calculated from current responses to voltage-ramps from representative whole-cell patch-clamp experiments on cells expressing TMEM175 wt (*A*) and TMEM175 H57Y (*B*). Pipette solution contained 140 mM K^+^ and was buffered with 50 mM HEPES to pH 7.4. [K^+^]_ex_ and pH_ex_ were changed during the experiments as indicated above the traces. pH_ex_ was buffered with 50 mM HEPES (pH_ex_ = 7.4) or 50 mM citrate (pH_ex_ = 4.7). For reducing [K^+^]_ex_ from 140 to 14 mM potassium was replaced by equimolar amount of NMDG^+^. (*C*) Chord conductance at −120 mV of GFP (ctrl), TMEM175 wt-, and TMEM175 H57Y-expressing HEK293 cells calculated from recordings as in (*A*) and (*B*) with [K^+^]_ex_ = 140 mM and pH_ex_ = 7.4 (time point *a*) or pH_ex_ = 4.7 (time point *c*). (*D*) Peak E_rev_ measured after pH_ex_-jump from 7.4 to 4.7 in [K^+^]_ex_ = 140 mM. For TMEM175 H57Y peak E_rev_ = steady-state E_rev_ (time point *c*). (*E*) ∆E_rev_ obtained between time points *c* and *d* in (*A*) and (*B*) following reduction of [K^+^]_ex_ from 140 mM to 14 mM. Values were only taken from recordings in which E_rev_ had reached a stable ΔpH at time point *c*. (*F*) Ratios of H^+^- and K^+^-permeabilities (P_H+_/P_K+_) at pH_ex_ = 4.7 calculated from E_rev_ and ΔE_rev_ values in (*D*) and (*E*) using Eqs. **1** and **2**, respectively. (*G*) Ratios of H^+^ and K^+^ permeabilities of TMEM175 wt at pH_ex_ = 7.4 [calculated from E_rev_ values at time point *f* in (*A*)], pH_ex_ = 6.1 (calculated from ΔE_rev_ between time points *b* and *a* in *SI Appendix*, Fig. S4*C*) and pH_ex_ = 4.7 [pooled values for TMEM175 wt from (*F*)]. Bars in (*C*), (*F*), and (*G*) represent geometric mean ± geometric SD. Statistical comparisons within different groups in (*C*) (separated by dotted lines) were performed using a ratio paired *t* test. Statistical comparisons between different groups in (*C*), (*F*), and (*G*) were performed using a lognormal ordinary one-way ANOVA with Šídák’s multiple comparisons test. Bars in (*D*) and (*E*) represent arithmetic mean ± SD. Statistical comparisons in (*D*) and (*E*) were performed using an unpaired *t* test with Welch’s correction.

To investigate the contribution of H57 to TMEM175 conductance, H^+^/K^+^-selectivity, and gating in more detail, we expressed TMEM175 wt and its H57Y mutant in HEK293 cells and performed whole-cell patch-clamp experiments similar to those shown in Figs. 1 and 2. For analyzing the relationship between H^+^- and K^+^-permeabilities under different external pH conditions, both the pH- and [K^+^]-gradient were varied during the recordings (Fig. 4 *A* and *B*). The conductance of TMEM175 H57Y-expressing cells in symmetrical solutions with 140 mM K^+^ and pH 7.4 was about 7 times lower than the conductance of TMEM175 wt-expressing cells, and only 2.5 times (and not significantly) higher than the conductance of control cells (Fig. 4*C*). However, the former cells still reacted to a jump in pH_ex_ to 4.7 with an increase in conductance (Fig. 4 *B* and *C*) and a shift of E_rev_ to positive values in comparison with control cells (Fig. 4 *B* and *D*). Interestingly, this shift was not transient but stabilized at a value of +32.9 ± 4.5 mV (n = 11); this is significantly lower than the highest peak E_rev_ values observed in TMEM175 wt-expressing cells (+52.5 ± 3.2 mV, n = 5) (Figs. 2*E* and 4*D*).

With these well-defined E_rev_ values we determine from Eq. **1** for the wt channel at pH_ex_ 4.7 a P_H+_/P_K+_ ratio of 50.7 · 10^3^ ×/÷ 0.062, a value similar to those reported previously (4, 5). The high P_H+_/P_K+_ value reduces to 19.2 · 10^3^ ×/÷ 0.112 in the mutant suggesting that the H57Y mutation increases the preference for transport of K^+^ ions by a factor of 2.6. This relative increase in K^+^-selectivity of the mutant was confirmed by the change in E_rev_ caused by a 10-fold reduction of [K^+^]_ex_: Because of a higher contribution of K^+^-current, the latter shifted E_rev_ by −8.5 ± 1.2 mV (n = 8) in TMEM175 H57Y-expressing cells, which is more negative than in cells expressing the wt channel (−3.8 ± 0.9 mV, n = 4) (Fig. 4*E*).

Based on the GHK-model (Eq. **2**), the P_H+_/P_K+_ ratios calculated from these ΔE_rev_ values do not differ from those calculated from peak E_rev_ values (Fig. 4*F*), which once again confirms the reduced H^+^/K^+^-selectivity of the H57Y mutant. It further demonstrates that the P_H+_/P_K+_ ratio is insensitive to [K^+^]_ex_ and that H^+^-conductance is mechanistically independent of K^+^.

Experiments in Fig. 4*A* also allow the calculation of P_H+_/P_K+_ under neutral conditions: in TMEM175 wt-expressing cells, E_rev_ settles at −37.1 ± 2.5 mV (n = 15), which is about 20 mV away from E_K+_ and can be translated into a P_H+_/P_K+_ ratio of 490 · 10^3^ ×/÷ 0.105. In line with the P_H+_/P_K+_ value calculated from experiments with pH_ex_ 6.1 (*SI Appendix*, Fig. S4*C*), which is 87.1 · 10^3^ ×/÷ 0.134 (n = 17), the data in Fig. 4*G* imply a pH dependency of the channel’s selectivity. Reduction of pH_ex_ from 7.4 to 4.7 lowers the H^+^/K^+^ selectivity of the wt channel about 10-fold (Fig. 4*G*).

The reduced K^+^ and H^+^ conductivity and the altered P_H+_/P_K+_ ratio in the H57Y mutant channel show that H57, as a pH-sensitive element of the luminal salt bridge network, plays a critical role in gating and cation selectivity of TMEM175. According to this model the open state should be stabilized by substituting H57 with a positively charged amino acid. This prediction however is not met by our finding that the H57K mutant generates no measurable current (*SI Appendix*, Fig. S10). A possible explanation is that differences in steric properties between the lysine residue and the protonated histidine may interfere with the correct formation of the luminal salt bridge network. The role of H57 as a functional element in a salt bridge network is in line with a previously identified functional significance of some of these amino acids (4, 5) and further supported by our finding that neutralization of the salt bridge partners D279 and E282 leads to a loss of measurable ion channel activity (*SI Appendix*, Fig. S10).

### Conclusion.

The present experiments underpin the complex structure/function correlates in the lysosomal cation channel TMEM175, with evidence for pH-dependent impacts on gating and H^+^/K^+^-selectivity. TMEM175 conducts a mixed H^+^/K^+^ current in neutral as well as in acidic pH. At neutral pH, this conductance is low, with a high preference for H^+^ over K^+^. However, since the K^+^ concentration is one million times higher than the H^+^ concentration under these conditions, the channel conducts mostly K^+^ ions at neutral pH. Acidification of the lysosomal lumen triggers two functionally independent and temporally uncoupled mechanisms: It instantly activates a gate, which augments the channel’s K^+^-conductance and a delayed H^+^-conductance. The resulting high H^+^ influx into the cytosol causes, under experimental conditions, a rapid erosion of the pH-gradient across the membrane—even in the presence of high buffer concentrations.

H57 located on the luminal side of the channel is one element of a pH-sensor with gating function. This titratable amino acid with a pK_A_ value of ~7 is part of a salt bridge network at the luminal pore entrance that stabilizes the channel’s open state. Neutralization of H57 or its anionic salt bridge partners consequently results in great reduction or suppression of acid-induced TMEM175 conductance.

pH-dependent gating of TMEM175 via protonation of H57 not only increases TMEM175 conductance for K^+^ and H^+^, but also changes their relative permeabilities, suggesting that the permeation mechanisms for K^+^ and H^+^ are closely interconnected. Since the H57Y substitution does not completely eliminate neither H^+^ nor K^+^ conductance, H57 has a sensory function but is not an essential part of the H^+^ or K^+^ permeation pathway.

The biophysical properties of the TMEM175 channel with its mutual interplay between H^+^- and K^+^- conductance and pH-sensitivity are in good agreement with the channel’s proposed role in assuring ion homeostasis in lysosomes (18). The channel can integrate the pH status in the lysosome lumen as well as the K^+^ concentration on both sides of the organelle membrane. In this way it can modulate both the amount of current as well as its ionic flavor according to the physiological demand. It is tempting to speculate that the delay between acid triggered K^+^ and H^+^ conductance serves as a “low pass filter” with the effect that channel activity is only upregulated by sustained luminal hyperacidification and not during brief pH-fluctuations which may frequently occur in the small organelle volume.

## Materials and Methods

### Cloning.

The expression vector pEGFP-N2 was cut with NotI and XhoI to remove the EGFP gene. The sequence coding for human TMEM175 was amplified from pcDNA3.1-hTMEM175_STOPSTOP (6) using primer pair 1 (Table 1) and inserted into the pEGFP-N2 backbone downstream of the CMV promoter sequence via Gibson assembly using the NEBuilder® HiFi DNA Assembly Master Mix (NEB, Ipswich, MA). Reaction products were transformed into chemically competent DH5α *Escherichia coli* by heat shock and positive clones were selected on LB agar plates containing 25 µg/mL kanamycin. Plasmid DNA was isolated from overnight cultures using the ZymoPURE Plasmid Miniprep Kit (Zymo Research Europe GmbH, Freiburg, Germany). Site-directed mutagenesis (SDM) was performed to generate the p.H57Y, p.H57K, and p.D279N/E282Q substitutions in TMEM175 using primer pair 2, 3, and 4 (Table 1), respectively. Success of cloning and SDM was confirmed by Sanger sequencing (Microsynth Seqlab, Göttingen, Germany).

**Table 1.** Primers used for cloning and SDM

Pair		Sequence (5′->3′)
1	Forward	CCGGACTCAGATCTCGAGATGTCCCAGCCCCGGACCCC
	Reverse	GATCTAGAGTCGCGGCCGCCTAGCAGGGGGCAGGGAGGAG
2	Forward	CTGCCTGTGACCTACACGGAGATCTCC
	Reverse	GGAGATCTCCGTGTAGGTCACAGGCAG
3	Forward	CCTGTGACCAAGACGGAGATC
	Reverse	GATCTCCGTCTTGGTCACAGG
4	Forward	CATCCTGAACATCTGCCAAGACAACGTCCCGG
	Reverse	GTTGTCTTGGCAGATGTTCAGGATGAGAAGCG

### Cell Culture.

For functional expression of TMEM175 and TMEM175 mutants, HEK293 cells were transfected 16 to 24 h before patch-clamp experiments using TransfeX™ Transfection Reagent (LGC Standards GmbH, Wesel, Germany) according to the manufacturer’s instructions. HEK293 cells were grown at 37 °C in a humidified 95% air/5% CO_2_ incubator in Dulbecco’s Modified Eagle Medium (DMEM; Thermo Fisher Scientific, Waltham, MA) supplemented with 10% v/v heat-inactivated fetal bovine serum, 100 U/mL penicillin G, 100 μg/mL streptomycin sulfate and 2 mM L-glutamine (all from Invitrogen). After reaching approximately 80% confluence, cells were (co-)transfected in a 35 mm petri dish with 1 µg of the plasmid carrying the gene of interest and (if necessary) 1 µg of empty pEGFP-N2 vector to be able to identify transfected cells via eGFP fluorescence.

### Plasma Membrane Patch-Clamp Electrophysiology.

On the day of the experiment, transfected HEK293 cells were separated by trypsinization, seeded at low density on 15 mm coverslips, and then incubated for 2 to 4 h to allow adherence of cells to the coverslip. Coverslips were transferred to a perfusion chamber filled with bath solution and placed on the stage of an inverted microscope. Transfected cells were identified by the fluorescence of coexpressed eGFP. Patch-clamp experiments were performed at room temperature (20 to 25 °C) with an EPC-9 amplifier (HEKA Elektronik, Lambrecht, Germany) or a LM-PC patch-clamp amplifier (List-Medical, Darmstadt, Germany) combined with a LIH 8 + 8 AD/DA interface (HEKA Elektronik) in the whole-cell configuration. Patch-pipettes were pulled from borosilicate glass capillaries (34500-99 Kimble Kimax from DWK Life Sciences, Milville, NJ, or TW150-4 from World Precision Instruments, Friedberg, Germany) using a single stage glass microelectrode puller (PP-830, Narishige Group, Tokyo, Japan) or a DMZ Universal Puller (Zeitz, Munich, Germany) resulting in 3 to 5 MΩ resistances. Capillaries were coated with Sigmacote® (Merck KgaA, Darmstadt, Germany) and baked after pulling at 70 °C for 15 to 45 min. Currents were recorded with a 2.9 kHz low-pass Bessel filter and sampled with a frequency of 20 kHz.

To determine the time-dependent activation of TMEM175 after pH activation a symmetrical ramp protocol was used. A holding potential of +120 mV is held for 25 ms followed by a voltage-ramp for 150 ms to a holding potential of −120 mV for another 25 ms. Afterward the voltage was continuously increased to +120 mV over 150 ms.

Free running membrane potentials were measured in current-clamp mode at zero current. Here, the “Gentle CC-Switch” option in PatchMaster was deactivated, so that the holding potential is not retained when switching from voltage-clamp to current-clamp, but changes directly to 0 pA. Liquid junction potentials (LJPs) were calculated using JPCalcWin (UNSW, Sydney, NSW, Australia) and subtracted post recording. Data were stored with PatchMaster (HEKA Elektronik, Lambrecht, Germany) and analyzed with FitMaster (HEKA Elektronik, Lambrecht, Germany).

Standard pipette solution contained (in mM): 137.5 potassium methanesulfonate (KCH_3_SO_3_; K-MS), 10 tetraethylammonium hydroxide (TEA-OH), 5 cesium methanesulfonate (CsCH_3_SO_3_; Cs-MS), 2.5 MgCl_2_, 5 4-(2-hydroxyethyl)-1-piperazineethanesulfonic acid (HEPES), 2.5 KOH, 1 ethylene glycol-bis(β-aminoethyl ether)-N,N,N′,N′-tetraacetic acid (EGTA).

Standard bath solution with pH 7.4 contained (in mM): 137.5 K-MS, 10 TEA-OH, 5 Cs-MS, 0.5 MgCl_2_, 2 CaCl_2_, 5 HEPES, 2.5 KOH. Standard bath solution with pH 4.7 contained in mM: 137.5 K-MS, 10 TEA-OH, 5 Cs-MS, 0.5 MgCl_2_, 2 CaCl_2_, 2.5 potassium acetate (KOAc), and 2.5 acetic acid (HOAc).

Bath solution with 14 mM K+ and pH 7.4 contained (in mM): 11.5 K-MS, 126 N-Methyl-D-glucamine (NMDG),10 TEA-OH, 5 Cs-MS, 0.5 MgCl_2_, 2 CaCl_2_, 5 HEPES, 2.5 KOH. The corresponding bath solution with pH 4.7 contained (in mM): 11.5 K-MS, 126 NMDG, 10 TEA-OH, 5 Cs-MS, 0.5 MgCl_2_, 2 CaCl_2_, 2.5 KOAc, and 2.5 HOAc.

For experiments with 50 mM pH buffer the pipette solution contained in (mM): 115 K-MS, 10 TEA-OH, 5 Cs-MS, 2.5 MgCl_2_, 50 HEPES, 25 KOH, 1 EGTA. The corresponding bath solution at pH 7.4 contained (in mM): 115 K-MS, 10 TEA-OH, 5 Cs-MS, 0.5 MgCl_2_, 2 CaCl_2_, 50 HEPES, 25 KOH. The bath solution with pH 4.7 and 50 mM acetate contained (in mM): 115 K-MS, 10 TEA-OH, 5 Cs-MS, 0.5 MgCl_2_, 2 CaCl_2_, 25 KOAc, and 25 HOAc. The bath solution with 50 mM citrate, 140 K^+^, and pH 4.7 contained (in mM): 40 K-MS, 100 KOH, 10 TEA-OH, 5 Cs-MS, 0.5 MgCl_2_, 2 CaCl_2_, 50 citric acid. The corresponding bath solution with 14 mM K^+^ contained (in mM): 14 K-MS, 126 NMDG, 10 TEA-OH, 5 Cs-MS, 0.5 MgCl_2_, 2 CaCl_2_, 50 citric acid. The bath solution with pH 6.1 contained (in mM): 115 K-MS, 10 TEA-OH, 5 Cs-MS, 0.5 MgCl_2_, 2 CaCl_2_, 50 2-(*N*-morpholino)ethanesulfonic acid (MES), 25 KOH.

The pH of all solutions was adjusted with methanesulfonic acid (MSA) to 4.7, 6.1, or 7.4. The osmolarity of pipette and bath solutions was adjusted to 290 mOsmol/kg and 300 mOsmol/kg, respectively, with D-mannitol.

### Endolysosomal Patch-Clamp Electrophysiology.

Whole-endolysosomal electrophysiology was performed in isolated enlarged endosome/endolysosome vacuoles using a modified patch-clamp method as described previously (19). HEK293 cells were plated onto poly-L-lysine (Sigma)-coated glass coverslips, grown over night and transiently transfected for 16 to 24 h with plasmids using TurboFect (Thermo Fisher Scientific, Waltham, MA) according to the manufacturer’s instructions. Cells were then treated for >12 h with 1 µM apilimod (Merck KgaA, Darmstadt, Germany), a lipophilic polycyclic triazine that selectively increases the size of endosomes/endolysosomes. Large vacuoles/endolysosomes were observed in most apilimod-treated cells. Transfected cells were identified by the fluorescence of coexpressed eGFP. Glass pipettes for recording were pulled from borosilicate capillaries (DWK Life Sciences, Milville, NJ) and polished and had a resistance of 4 to 8 MΩ. Patch-clamp experiments were performed at room temperature (20 to 25 °C) with an EPC-10 amplifier (HEKA Elektronik, Lambrecht, Germany) and PatchMaster acquisition software (HEKA) in the whole-endolysosome configuration on manually isolated enlarged vacuoles. In brief, a patch pipette was pressed against a cell and quickly pulled away to rapture the cell membrane. This allowed to manually isolate enlarged endolysosomes into the recording chamber and identify TMEM175-expressing vacuoles by monitoring eGFP fluorescence. After formation of a gigaseal between the patch pipette and an enlarged endolysosome, capacitance transients were compensated. Voltage steps of several hundred millivolts with millisecond duration(s) were then applied to establish the whole-endolysosome configuration.

The pipette solution at pH 7.4 contained in (mM): 137.5 K-MS, 10 TEA-OH, 5 Cs-MS, 0.5 MgCl_2_, 1.8 CaCl_2_, 5 HEPES, 2.5 KOH. pH and osmolarity were adjusted to 7.4 with MSA and 290 mOsmol/kg with D-mannitol, respectively. The pipette solution at pH 4.7 contained in mM: 137.5 K-MS, 10 TEA-OH, 5 Cs-MS, 0.5 MgCl_2_, 1.8 CaCl_2_, 2.5 KOAc, and 2.5 HOAc. pH and osmolarity were adjusted to 4.7 with MSA and 300 mOsmol/kg with D-mannitol, respectively. Standard bath solution contained in mM: 137.5 K-MS, 10 TEA-OH, 5 Cs-MS, 2.5 MgCl_2_, 5 HEPES, 2.5 KOH, 1 EGTA. pH and osmolarity were adjusted to 7.4 with MSA and 300 mOsmol/kg with D-mannitol, respectively.

To measure steady-state currents a step protocol was used. After holding 0 mV for 100 ms, test pulses starting from −120 mV were applied with a voltage increment of 20 mV for 200 ms. A holding potential of 0 mV was then clamped for further 100 ms.

### Data Analysis and Statistics.

Data were exported using PatchMaster and FitMaster (HEKA Elektronik, Lambrecht, Germany) and sorted as well as analyzed in Excel (Microsoft; Redmond, Washington, DC) and MATLAB (MathWorks; Natick, MA). Statistical analyses were carried out in GraphPad Prism (GraphPad Software, Boston, MA).

Data are presented as mean ± SD or geometric mean ×/÷ geometric SE from at least three independent experiments. The employed statistical tests are indicated in the figure captions. *P*-values are reported as follows: ns: not significant, *P < 0.05, **P < 0.01, ***P < 0.001, ****P < 0.0001.

Permeability ratios P_H+_/P_K+_ were calculated from absolute values of measured reversal potentials (E_rev_) or changes in E_rev_ (ΔE_rev_) using the Goldman–Hodgkin–Katz (GHK) model:

[1] $$\frac{P_{H^{+}}}{P_{K^{+}}}=\frac{{\left[K^{+}\right]}_{\mathrm{ex}}-{\left[K^{+}\right]}_{\mathrm{in}}∙e^{\frac{F}{\mathrm{RT}}∙E_{\mathrm{rev}}}}{10^{-{\mathrm{pH}}_{\mathrm{in}}}∙e^{\frac{F}{\mathrm{RT}}∙E_{\mathrm{rev}}}-10^{-{\mathrm{pH}}_{\mathrm{ex}}}},$$

[2] $$\frac{P_{H^{+}}}{P_{K^{+}}}=\frac{{\left[K^{+}\right]}_{ex,2}-{\left[K^{+}\right]}_{ex,1}∙e^{\frac{F}{\mathrm{RT}}∙\Delta E_{\mathrm{rev}}}}{10^{-{\mathrm{pH}}_{\mathrm{ex}}}∙e^{\frac{F}{\mathrm{RT}}∙\Delta E_{\mathrm{rev}}}-10^{-{\mathrm{pH}}_{\mathrm{ex}}}},$$

with pH_in_ being the pH of the internal solution, pH_ex_ the pH of the external solution, [K^+^]_in_ the K^+^ concentration of the internal solution, [K^+^]_ex_ the K^+^ concentration of the external solution, F the Faraday constant, R the gas constant and T the absolute temperature in Kelvin. [K^+^]_ex,1_ and [K^+^]_ex,2_ in Eq. **2** are the K^+^ concentrations before and after the [K^+^]-jump that caused ΔE_rev_. Eq. **2** was only used if E_rev_ and thus pH_in_ had reached a stable value before the [K^+^]_ex_-jump. Since all recording solutions contained 5 mM Cs^+^ and the P_Cs+_/P_K+_ ratio is close to 1 (5, 6), it was assumed that the K^+^ concentrations were 5 mM higher than the nominal adjusted K^+^ concentrations for the calculation of the P_H+_/P_K+_ ratios.

### SSME recordings.

SSME recordings of TMEM175 activity in native lysosomes from transiently transfected HEK293 cells were performed as described previously (17). Since generated current amplitudes in lysosomes from transiently transfected HEK293 were considerably lower than described for lysosomes from a stable TMEM175-expressing cell line (17), an in-well control was employed to record the background current for each sensor containing lysosomes with TMEM175. First, currents were recorded in the absence, and then in the presence of the TMEM175 blocker 4-AP (10 mM). The background current in presence of 4-AP was subtracted from that recorded in its absence to obtain the net TMEM175 current for all tested conditions.

Using the 4-AP protocol, we performed K^+^ concentration jumps in the range of 2 to 100 mM and pH-jumps starting at pH 7.6 to more acidic values with ΔpH between 0.2 and 3.

Buffers for K^+^ concentration jumps contained (in mM): 30 HEPES, 30 MES, 5 MgCl_2_, 90 NaCl (pH 7.6, NaOH); and were either supplemented with 300 mM NaCl (nonactivating solution, NA) or with 300 mM KCl (activating solution 1, A1). A1 was then diluted in NA to achieve activating solutions with final K^+^ concentrations between 2 mM and 100 mM (AX) for K^+^ concentration jump experiments. TMEM175 activity was stimulated via sequential solution exchange from NA to AX with decreasing K^+^ concentrations on the same sensor. TMEM175 activity was quantified from peak integrals (translocated charge) instead of peak currents, for a more accurate estimation of TMEM175 activity. This was necessary due to the overall smaller TMEM175 to background current ratio and the fact that the background current shows a negative amplitude (17). To derive the two slopes provided in *SI Appendix*, Fig. S7 *A* and *B*, reflecting the permeability ratio at different K^+^ doses (Fig. 3*K*), the data were fitted using a linear equation in the following two intervals: three data points between 2 and 8 mM and three data points between 32 and 80 mM.

Buffers for pH-jumps contained 30 mM HEPES, 30 mM MES, 140 mM NaCl, 5 mM MgCl_2_, and were titrated with NaOH to either pH 7.6 (nonactivating solution, NA) or to the desired pH between 4.6 and 7.4 (activating solutions AX). TMEM175 activity was stimulated via sequential solution exchange from NA to AX with increasing ΔpH on the same sensor. TMEM175 activity was measured using peak currents, as both background currents and those mediated by TMEM175 exhibit positive amplitudes (17). To derive the slope provided in *SI Appendix*, Fig. S7*B* reflecting the H^+^-permeability (Fig. 3*J*), the first 3 data points between ΔpH 0.2 and ΔpH 0.8 were fitted using a linear equation.

The pH dependence of K^+^ flux was determined using 50 mM K^+^ concentration jumps at different pH values on the same sensor. All measurement buffers contained (in mM): 30 HEPES, 30 MES, 5 MgCl_2_, 90 NaCl, and were titrated to 12 different pH values between 3.0 and 8.5, using either HCl or NaOH. Measurement buffers for each pH were then either supplemented with 50 mM NaCl (nonactivating solution, NA) or 50 mM KCl (activating solution, A). The measurement was initiated via solution exchange from NA to A at a given pH from alkaline to the acidic pH range. At the time point of the measurement, the given pH was symmetrically equilibrated, i.e., identical inside and outside of the lysosomes. This was controlled by incubating the sensor in the given pH for several minutes before the measurement. As the background current only depended on the dose used for the K^+^ concentration jump, an in-well control using 4-AP was not performed for each pH. Instead, the positive peak current related to net TMEM175 activity at a time point when the negative background current already decayed was used for analysis. This procedure was recently employed for the sodium/glucose cotransporter 1 (SGLT1) to dissect fast pre-steady-state currents from slow transport currents (20). The pH-dependent currents for each given sensor were normalized to the current recorded at pH 7.0, before averaging across sensors. To derive the pK_A_ value, the curve was fitted using a titration equation between pH 3 and pH 6.5 as detailed recently (17). As a comparison, we included the data for wt TMEM175 derived from Bazzone et al. where we employed a fit using a double titration equation, as two pK_A_ values were clearly visible.

### Structure Preparation and Modeling.

Human TMEM175 cryoEM-derived structures in the open (PDB Accession No.: 7UNL) and closed state (PDB Accession No.: 7UNM) were used as basis for modeling (7). To fill the existing gaps between residue 173 and residue 254 in both structures, the Alphafold2-multimer model as implemented by ColabFold was used to generate five individual structures each (21, 22). Those were energy-minimized according to the standard procedure in ColabFold (22).

The two structures with the lowest pLDDT and PAE scores were prepared for MD simulations. The alpha-helical parts of the structure show a pLDDT of about 70 while some unstructured parts are predicted with a pLDDT score below 70. Scores above 70 are commonly deemed acceptable, while lower pLDDTs are also associated with unstructured stretches in other studies (23, 24). As the main region of interest is located proximate to H57, a lower fidelity is acceptable in modeling.

Using CHARMM-GUI, the open and closed structures were embedded in POPC lipid bilayers, for a final KCl concentration of 150 mM.

Both the open and closed structure were prepared with H57 in the protonated and deprotonated form.

### MD Simulations.

Selected force fields as well as run parameters for the energy minimization, equilibration, and production MD simulations match a previously described procedure (25).

As sole deviation from this procedure, no electric field was applied during simulations.

In brief, the Amber99sb*-ILDN force field was combined with partially coarse-grained lipid parameters, constraints, and virtual sites for hydrogen atoms to allow for an increased time step of 4 fs (26–28). All simulations were run with Gromacs 2021.5 (29).

For all four investigated channel states, four individual production MD simulations were run, with a total simulation time of 3.5 µs per state (3 × 1 µs, 1 × 0.5 µs).

### Analysis of MD Trajectories.

Obtained MD trajectories were analyzed using Biotite (Version 0.41.0) together with Gromacs (v. 2021.5) analysis tools (29, 30). Various other Python libraries were used for general data analysis and visualization; a complete list of all used software and libraries is shown in *SI Appendix*, Table S1.

For all aggregated analysis plots shown in Fig. 3, the first 50 ns of each trajectory were discarded as additional equilibration time. In addition to 4.0 Å as an established interatomic distance threshold for salt bridges, 3.2 Å was chosen as a more conservative threshold, following standard parameters used in the VMD salt bridges plugin (31, 32).

## Supplementary Material

Appendix 01 (PDF)

## Data Availability

Modeled structures, MD input files as well as scripts required to reproduce analysis plots are available at https://doi.org/10.5281/zenodo.14719866 (33). Due to large file sizes, MD trajectories will be made available upon request. Electrophysiological data, log files and analysis files are available at: https://doi.org/10.5281/zenodo.18063925 (34).
