# Supplementary material for: Proton-selective conductance and gating of the lysosomal cation channel TMEM175
Source: Proc Natl Acad Sci U S A. 2026 Jan 14;123(3):e2503909123. doi: 10.1073/pnas.2503909123 (PMC12818570; doi:10.1073/pnas.2503909123)
Supplement: Supplementary file 1 — Appendix 01 (PDF) [file pnas.2503909123.sapp.pdf]

Supplementary Material

Proton selective conductance and gating of the lysosomal cation channel TMEM175

Tobias Schulze[a,b], Timon Sprave[a], Carolin Groebe[a,c], Jan Hendrik Krumbach[d], Magnus Behringer[d], Andre Bazzone[e], Rocco Zerlotti[e], Niels Fertig[e], Mike Althaus[f], Kay Hamacher[d], Gerhard Thiel[a,g], Christian Grimm[b,h,i,1], ✉ christian.grimm@med.uni-muenchen.de, and Oliver Rauh[a,f,1], ✉ oliver.rauh@h-brs.de

[a] Department of Biology, Membrane Biophysics, Technical University of Darmstadt, Darmstadt 64287, Germany

[b] Walther Straub Institute of Pharmacology and Toxicology, Endolysosomal Ion Channel Research, Faculty of Medicine, Ludwig-Maximilians-Universität, Munich 80336, Germany

[c] Institute of Pathophysiology, Physiology and Pathophysiology of Cortical Neuronal Networks, Biomedical Research Centre, University Medical Center of the Johannes Gutenberg University, Mainz 55128, Germany

[d] Department of Biology, Computational Biology & Simulation, Technical University of Darmstadt, Darmstadt 64287, Germany

[e] Nanion Technologies, Munich 80339, Germany

[f] Institute for Functional Gene Analytics, Department of Natural Sciences, Bonn-Rhein- Sieg University of Applied Sciences, Rheinbach 53359, Germany

[g] Department of Biosciences, University of Milan, Milan 20133, Italy

[h] Department of Pharmacology/Immunology, Infection and Pandemic Research, Fraunhofer Institute for Translational Medicine and Pharmacology, Munich 80799, Germany

[i] Department of Pharmacology, Faculty of Medicine, University of Oxford, Oxford OX1 3QT, United Kingdom

[1]To whom correspondence may be addressed. Email: ✉ christian.grimm@med.uni-muenchen.de or ✉ oliver.rauh@h-brs.de.

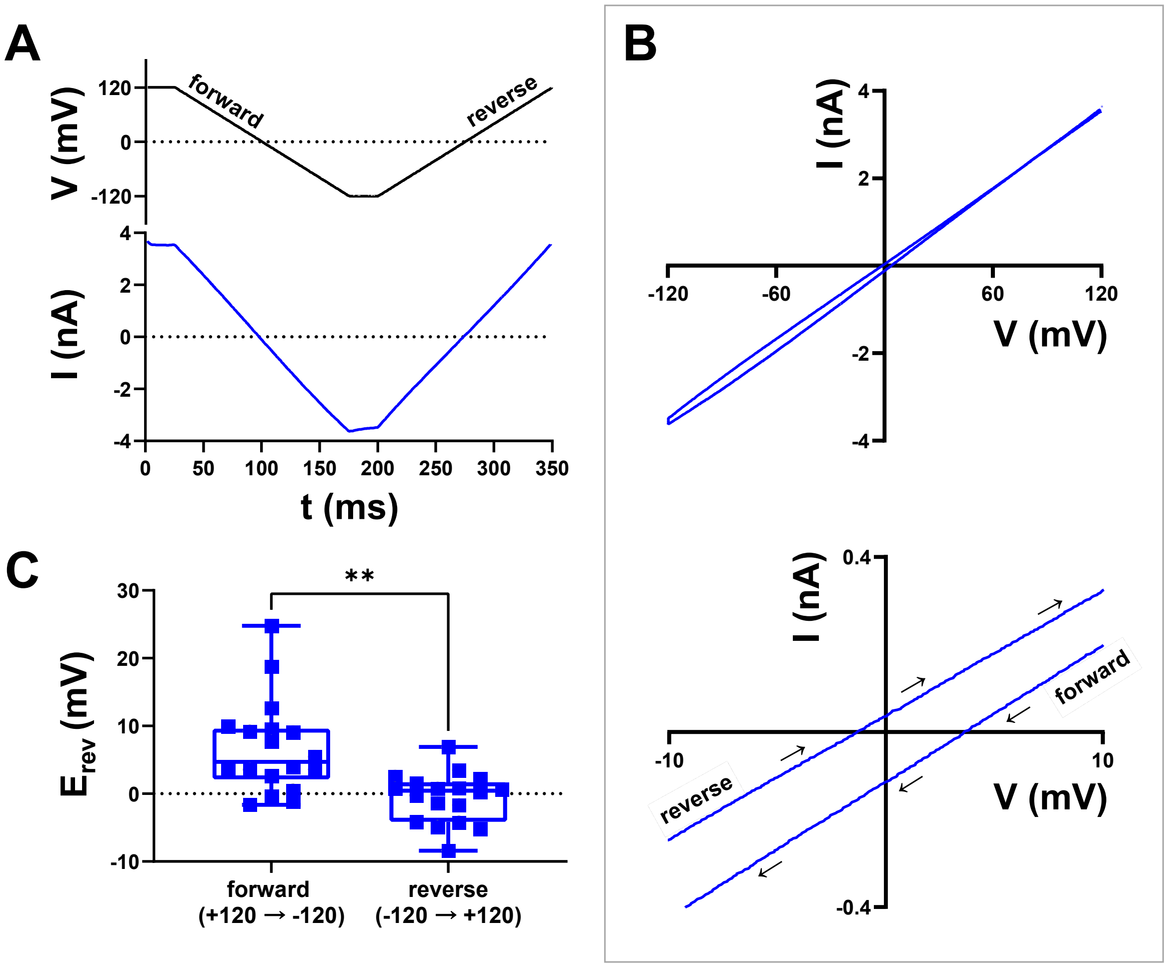

Fig.S1 Voltage-ramp evoked currents cause a shift in the current reversal potential of a TMEM175-expressing cell. (A) Voltage-ramp protocol (top) and representative current response (bottom) of a TMEM175-expressing cell, 3 min after the external buffer pH (pH_{ex}) was changed from 7.4 to 4.7. The experiment was performed as in Fig. 1. **(B)** Top: current response from experiments shown in (A), plotted against the applied voltage. Bottom: Magnification of currents to illustrate the shift of E_{rev} between forward and reverse ramps. The direction in which the voltage was changed during the forward and reverse ramps is indicated by arrows. **(C)** E_{rev} of the forward and reverse ramps from current responses as shown in (A). Paired two-tailed Student's t-test was used for statistical comparison.

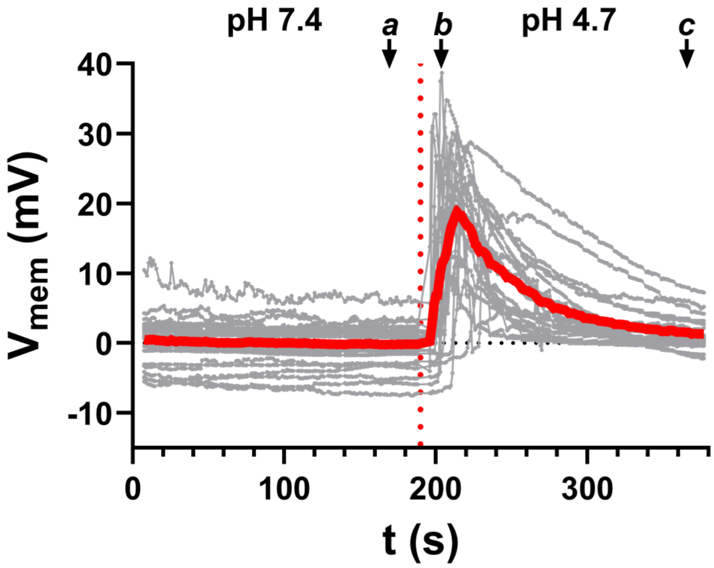

Fig.S2 Acidification of the bath solution induces transient shifts in free running membrane voltage to positive values in TMEM175-expressing cells. Individual (gray) and averaged (red) time-courses of V_{mem} of TMEM175-expressing HEK293 cells with symmetrical 140 mM K-MS; pH_{in} was 7.4. pH_{ex} is indicated at the top. Recordings were performed in the current-clamp mode. ΔV_{mem} values shown in Fig.1D were calculated from steady-state V_{mem} at time point *a* and peak V_{mem} at time point *b*.

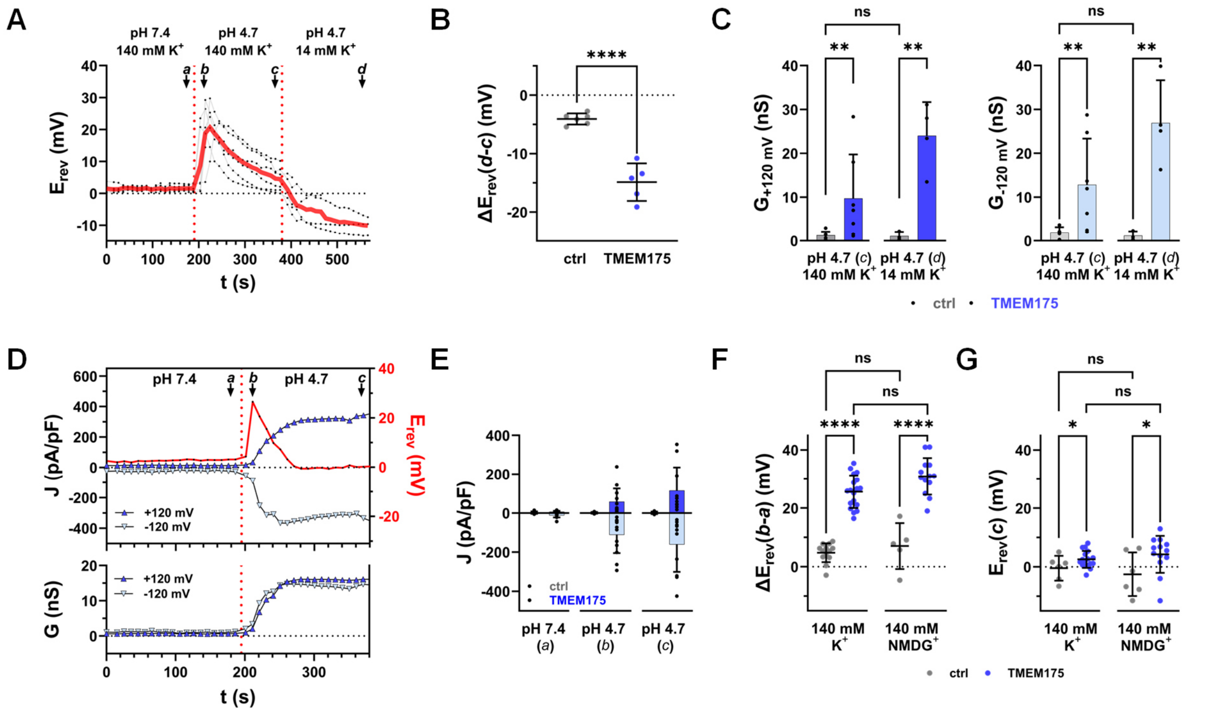

Fig.S3 The acid-induced transient shift of E_{rev} in TMEM175-expressing cells is independent of K^+-conductance. (A) Individual (black) and averaged time-courses (red) of E_{rev} of TMEM175-expressing cells derived from current responses to voltage-ramps as shown in Fig. 1. The internal 140 mM K-MS solution was buffered to pH 7.4. As indicated above the traces, pH_{ex} and external K^+ concentration were changed during the experiment from pH 7.4 to pH 4.7 and from 140 mM to 14 mM, respectively. **(B)** Change in E_{rev} in response to a reduction of external K^+ from 140 mM to 14 mM in control (gray) and TMEM175-expressing (blue) cells. E_{rev} values were taken at time points *c* and *d* from traces shown in panel (A). Statistical significance was tested by two-tailed, unpaired t-test with Welch's correction. **(C)** Chord conductance at +120 mV (left) and -120 mV (right) for control (gray) and TMEM175-expressing (blue) cells at time points *c* and *d* indicated in panel (A). **(D)** Representative time-courses of E_{rev}, current densities (upper graph) and chord conductance (lower graph) at ±120 mV of TMEM175-expressing cells, before and after pH_{ex} jump from 4.7 to 7.4 and in the absence of K^+ in internal and external solutions. Internal and external solutions contained 140 mM NMDG-MS; pH_{in} was 7.4. Values were calculated from current responses to voltage- ramps. **(E)** Current densities at ±120 mV of control (ctrl) and TMEM175-expressing cells at time points *a*, *b* and *c* as indicated in panel (D). **(F, G)** Maximal changes in E_{rev} in response to a pH jump from 7.4 to 4.7 **(F)** and in E_{rev} 3 min after the pH jump (time point *c* indicated in panel (D)) **(G)** of control (gray) and TMEM175-expressing (blue) cells. Recordings were performed in solutions containing symmetrical 140 mM K-MS (left) and 140 mM NMDG-MS (right). Bars in (B), (C) and (E-G) represent arithmetic mean ± SD; values from individual recordings are shown as closed circles. Statistical comparisons in (C), (F) and (G) were performed with unpaired two-way analysis of variance (ANOVA) followed by Tukey's multiple comparison test.

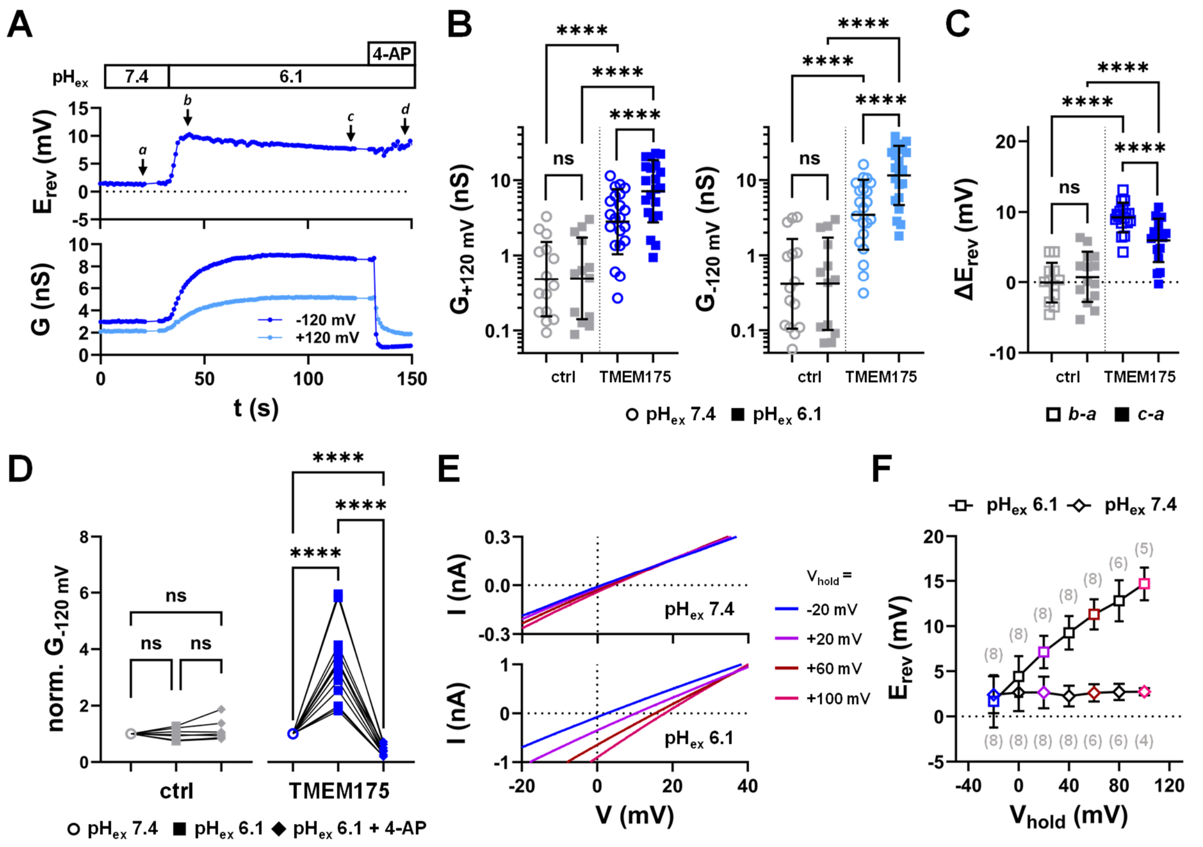

Fig.S4 Mild acidification of the luminal side causes an increase in TMEM175 conductance and a shift of E_{rev} towards E_{H+}. (A) Representative time-course of E_{rev} (upper graph) and corresponding chord conductance at ±120 mV (lower graph) of a TMEM175-expressing cell in response to a pH_{ex} jump from 7.4 (50 mM HEPES) to 6.1 (50 mM MES) and extracellular application of 1 mM 4-Aminopyridine (4-AP). Pipette solution contained 140 mM K^+ and was buffered with 50 mM HEPES to pH 7.4. **(B)** Chord conductance at +/-120 mV of GFP (ctrl) and TMEM175-expressing cells at pH_{ex} = 7.4 (time point *a* in (A)) and pH_{ex} = 6.1 (time point *c* in (A)). Bars represent geometric mean ± geometric SD. **(C)** Change in E_{rev} in response to pH_{ex} jumps from 7.4 to 6.1 in control and TMEM175-expressing cells. *a*, *b* and *c* refer to time points in (A). Bars represent arithmetic mean ± SD. Statistical comparisons within different groups (separated by dotted lines) were performed using a ratio paired t-test. Statistical comparisons between different groups were performed using an ordinary one-way ANOVA with Šídák's multiple comparisons test. **(D)** Normalized chord conductance at -120 mV of control (n = 8) and TMEM175-expressing cells (n = 13) measured at time points *a* (pH_{ex} = 7.4), *c* (pH_{ex} = 6.1) and *d* (pH_{ex} = 6.1 + 1 mM 4-AP). Values were normalized to corresponding conductance at pH_{ex} = 7.4.

Statistical comparisons within different groups (separated by dotted lines in (B,C)) were performed using a ratio paired t-test. Statistical comparisons between different groups in (B-D) were performed using a lognormal ordinary one-way ANOVA with Šídák's multiple comparisons test. **(E)** Representative current responses to 150 ms voltage ramps from +120 mV to -120 mV from different holding voltages (V_{hold}) between -20 mV and +100 mV at pH_{ex} = 7.4 and pH_{ex} = 6.1. **(F)** Arithmetic mean ± SD of E_{rev} values from (n) independent experiments as shown in (E), plotted as a function of V_{hold}.

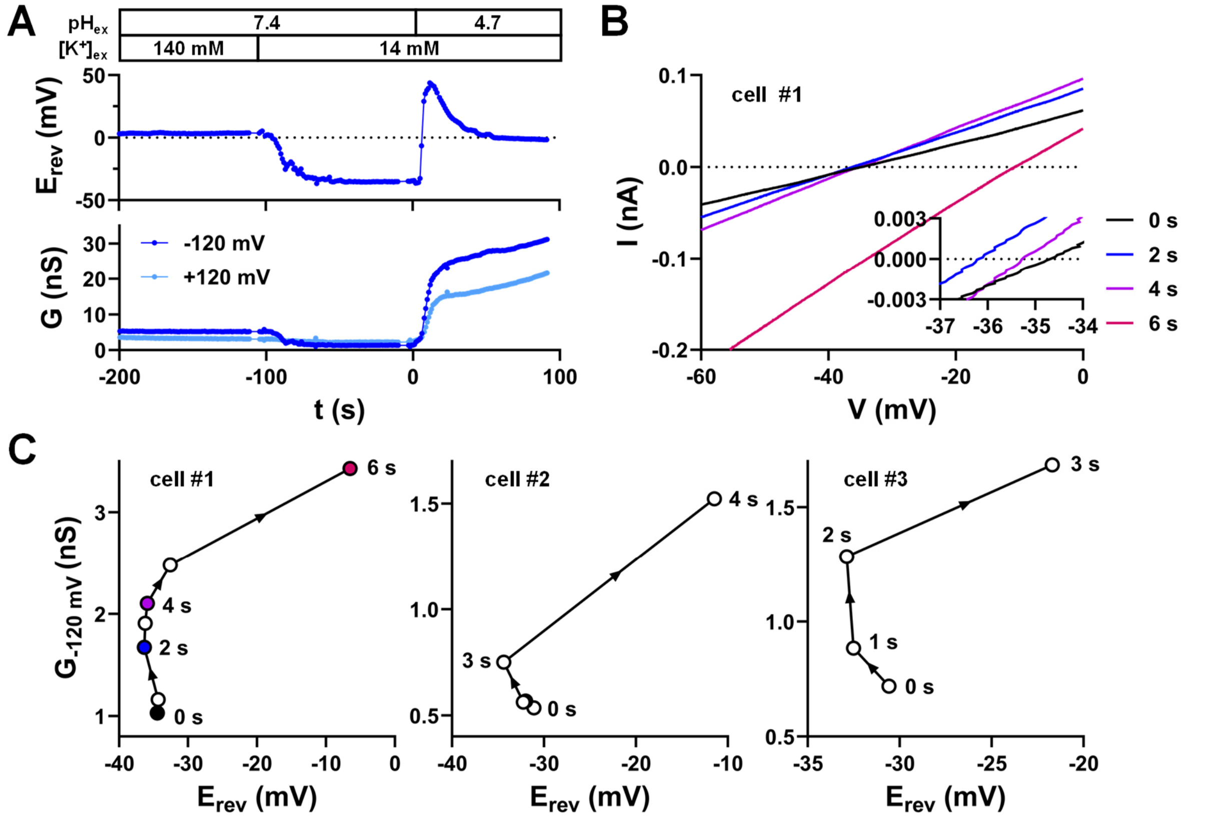

Fig.S5 Acidification of the luminal side activates K^+ conductance of TMEM175. **(A)** Time-courses of E_{rev} (upper graph) and chord conductance at ±120 mV (lower graph) calculated from current responses to voltage-ramps from a representative whole-cell patch-clamp experiment on a TMEM175-expressing cell. Pipette solution contained 140 mM K^+ and was buffered with 50 mM HEPES to pH 7.4. $[K^+]_{ex}$ and pH_{ex} were changed during the experiment as indicated above the traces. pH_{ex} was buffered with 50 mM HEPES (pH_{ex} = 7.4) or 50 mM citrate (pH_{ex} = 4.7). Potassium was replaced by equimolar amount of $NMDG^+$ to reduce $[K^+]_{ex}$ from 140 mM to 14 mM. t=0 corresponds to the time of the pH_{ex}-jump from 7.4 to 4.7. **(B)** Current responses from the experiment shown in (A) to voltage-ramps applied at indicated times after the pH_{ex}-jump from 7.4 to 4.7. Inset: magnification of current responses at 0, 2 and 4 s. **(C)** Conductance at -120 mV plotted against the corresponding E_{rev} values from three independent experiments as shown in (A). Values were calculated from current responses recorded immediately after the pH_{ex}-jump from 7.4 to 4.7 (t = 0). Data in the graph is derived from the experiment shown in (A) and (B).

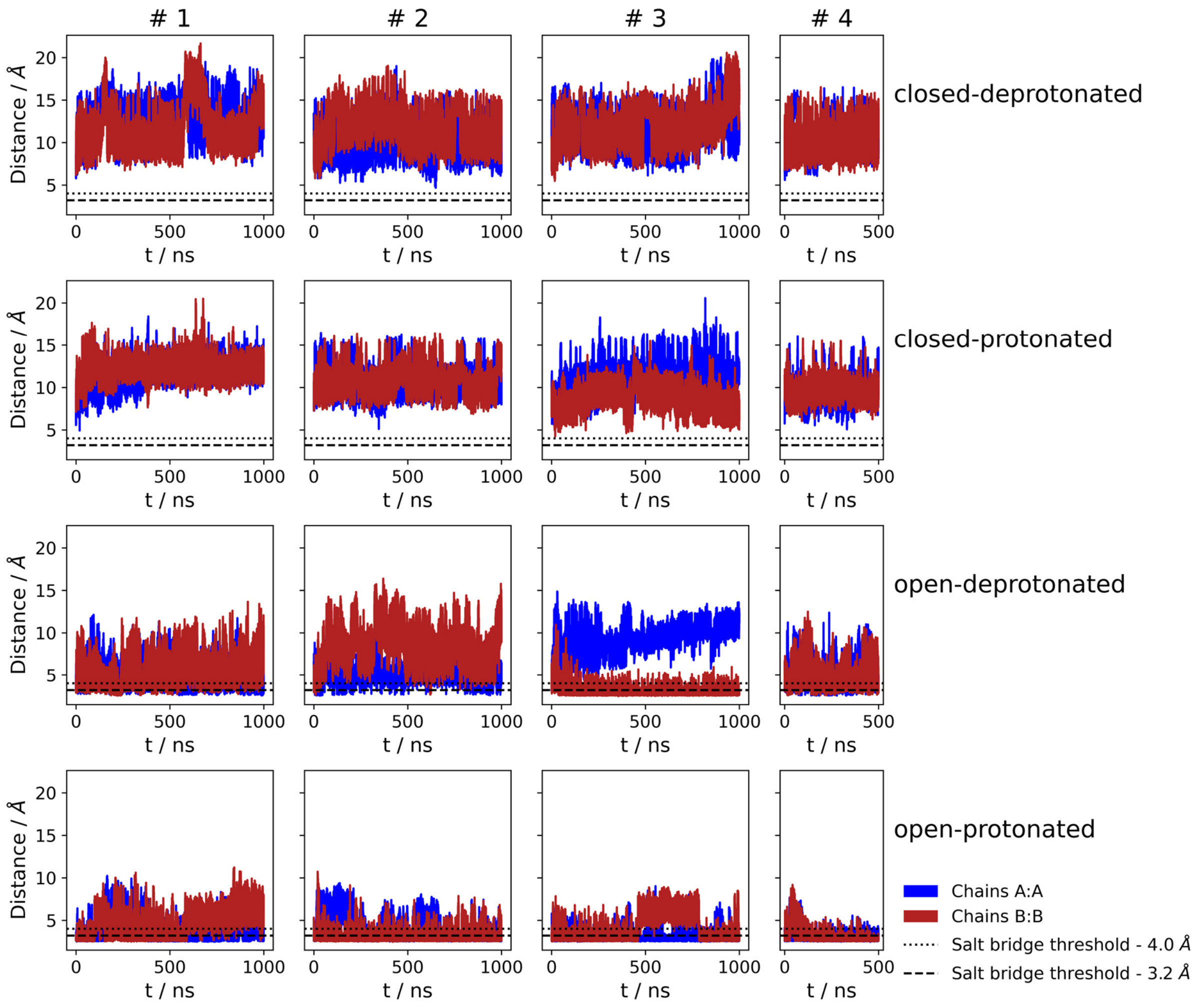

Fig.S6 H57-D279 (intra-chain) distance time series in MD trajectories of TMEM175. Shown data originate from four total MD replicas, run for 1 μs (#1-#3) and 0.5 μs (#4). These replicas were run for TMEM175 in each protonation state for the open and closed channel, respectively. Distances between H57 and D279 (intra-chain) are colored according to the subunit in which H57 resides (blue and red for chain A and B, respectively), with dotted/dashed lines for literature values for salt bridge distances (4 Å /3.2 Å).

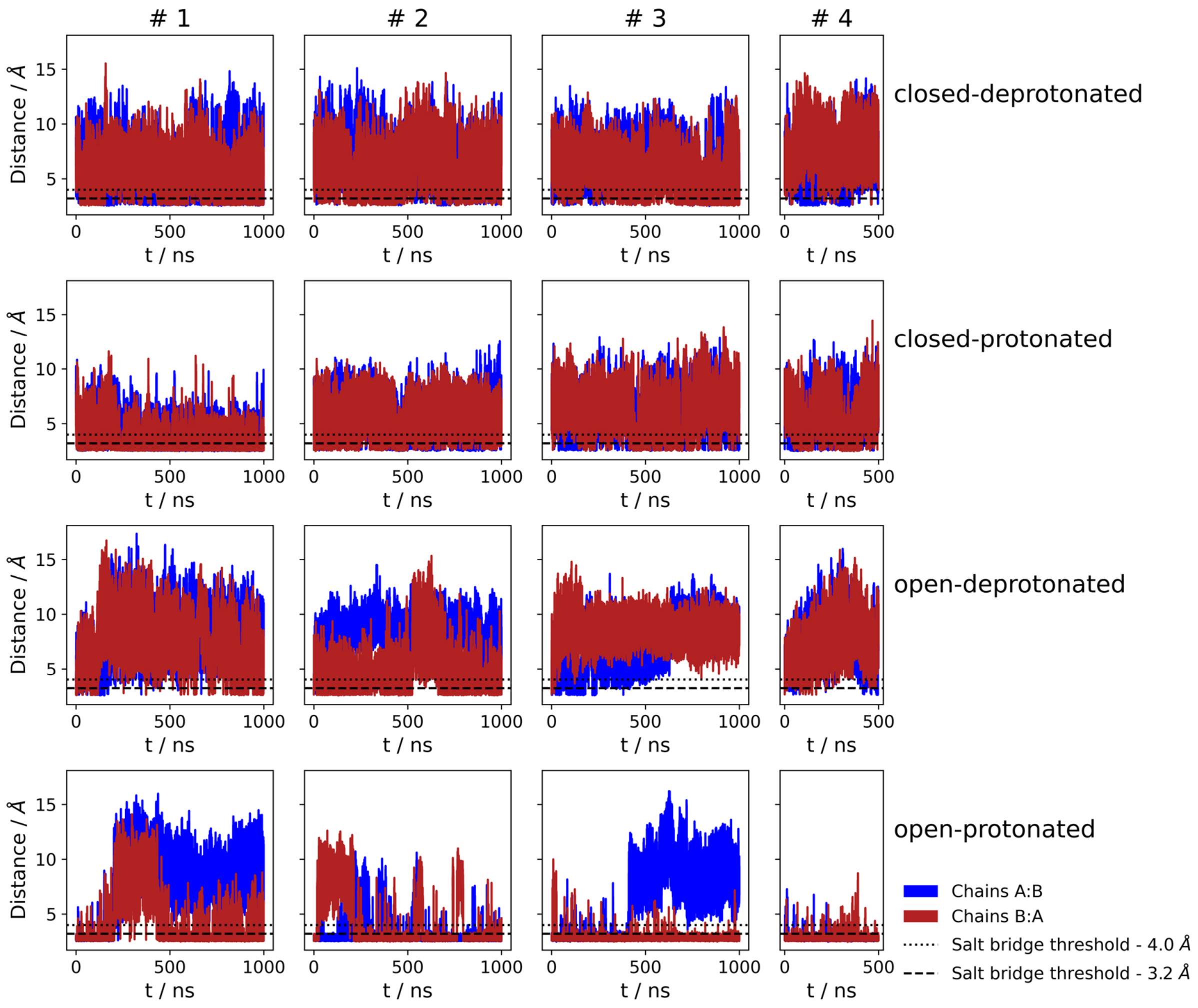

Fig.S7 H57-E282 (inter-chain) distance time series in MD trajectories of TMEM175. Shown data originate from four total MD replicas, run for 1 μs (#1-#3) and 0.5 μs (#4). These replicas were run for TMEM175 in each protonation state for the open and closed channel, respectively. Distances between H57 and E282 (inter-chain) are colored according to the subunit in which H57 resides (blue and red for monomer A and B, respectively), with dotted/dashed lines for literature values for salt bridge distances (4 Å /3.2 Å).

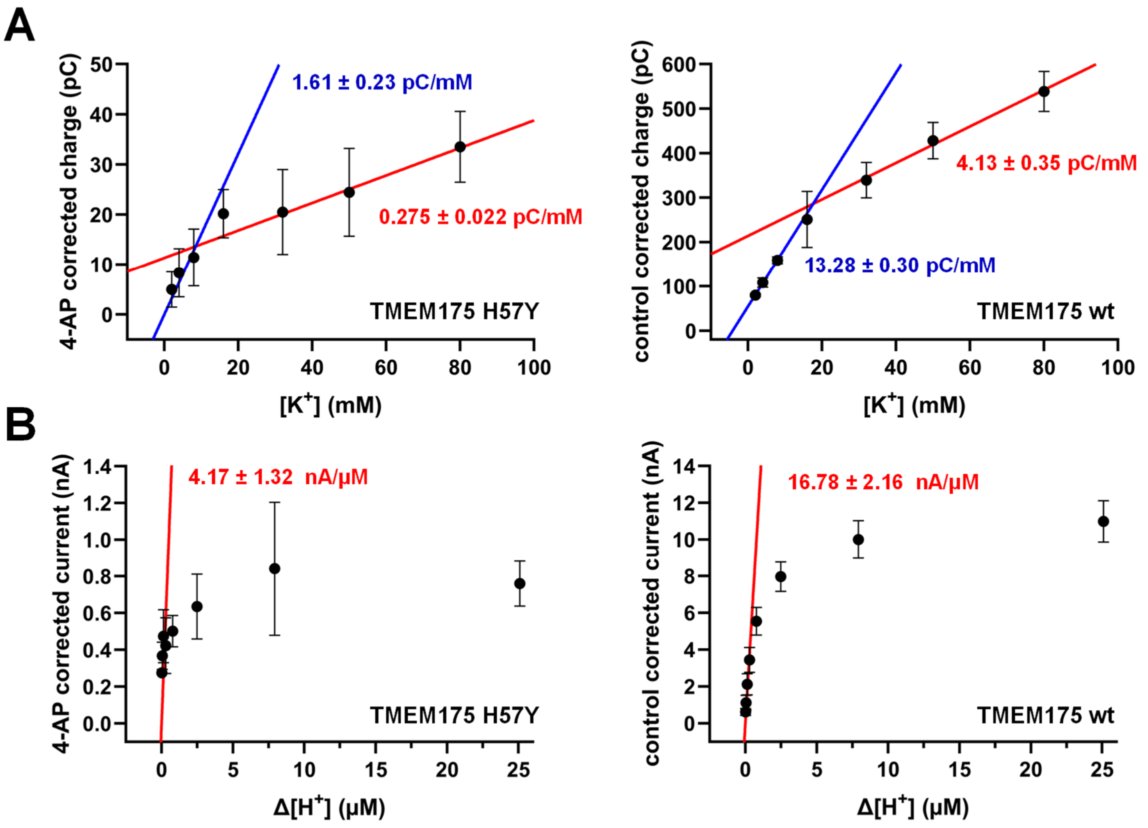

Fig.S8 Lysosomal SSME recordings reveal decreased K^+- and H^+-permeability in TMEM175 H57Y. Data for TMEM175 wt were adapted from (1) and represent arithmetic means ± SEM from n=8 different sensors. All data for the mutant represent arithmetic means ± SEM from n=5 independent sensors. **(A)** K^+ Flux Assay: K^+ flux was measured using sequential concentration jumps between 2 mM and 100 mM K^+ on the same sensor, driven solely by the chemical K^+ gradient. To account for opposing current directionalities between background and TMEM175 signals, charge analysis was performed by integrating the recorded transient currents. Background signals were recorded under identical conditions on the same sensor with 10 mM 4-AP and subtracted from the measurements in absence of 4-AP to obtain TMEM175 net signals. The dose-response curve exhibits a biphasic linear behavior: steep dependence below 10 mM and a flattened dependence above 30 mM, consistent with TMEM175 wt behavior. **(B)** H^+ Flux Assay: H^+ flux was measured using sequential pH-jumps starting at pH 7.6, with ΔpH ranging from 0.2 to 3.0 units toward acidic conditions. The pH-gradient drove H^+ influx, and peak current values were used for analysis due to the consistent directionality of currents from both background and TMEM175 signals. Background correction was performed as in (A). The resulting dose-response curve displays hyperbolic saturation kinetics, matching TMEM175 wt.

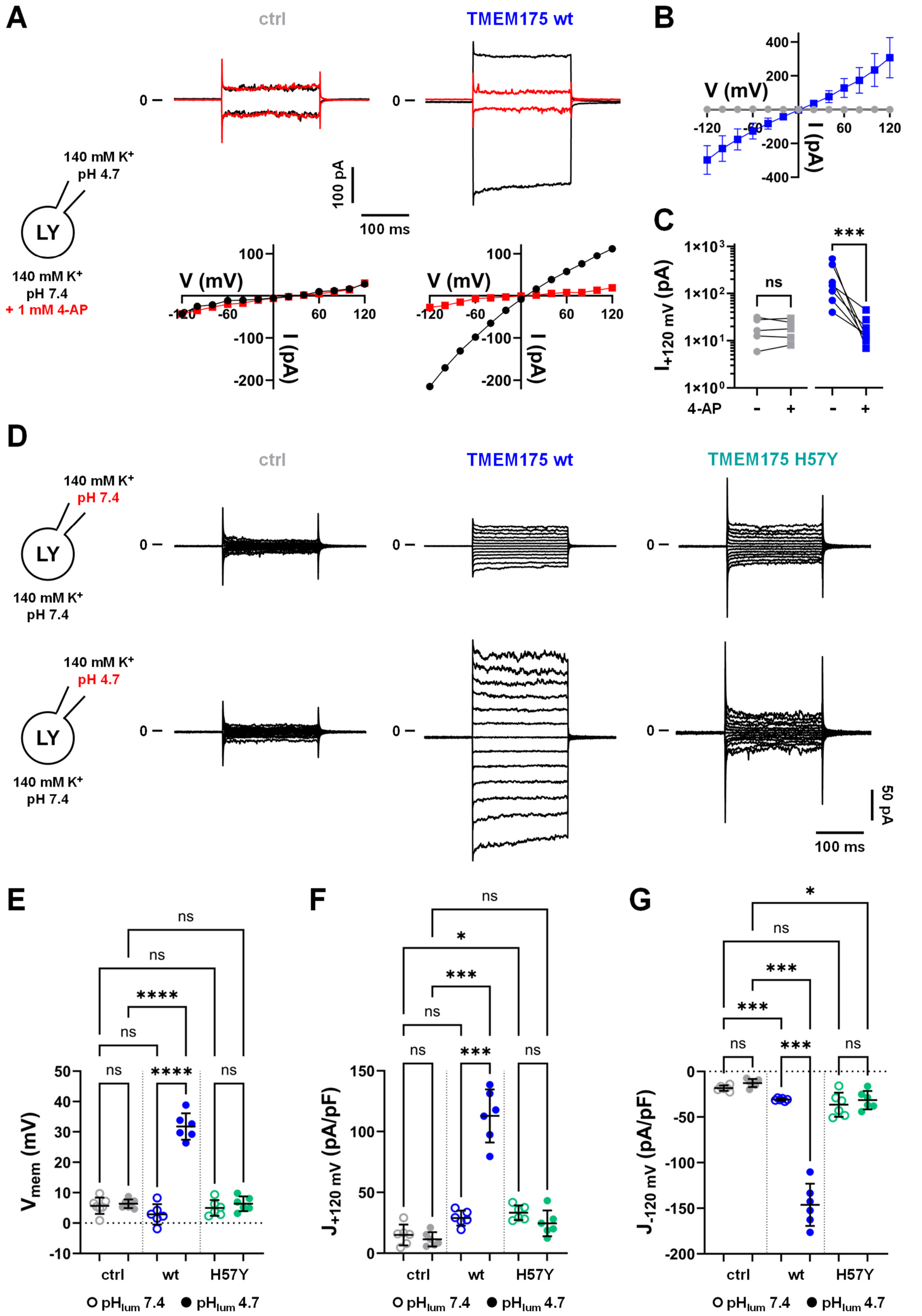

Fig.S9 H57Y mutation suppresses ion channel activity of TMEM175 in lysosomes. (A) Representative current responses of lysosomes (LY) from empty vector transfected (ctrl) and TMEM175-expressing HEK293 cells to test potentials of -

120 mV and +120 mV in the absence (black) and presence (red) of 1 mM 4-Aminopyridine (4-AP). Lysosomes were initially bathed in a buffer with pH 7.4 and 140 mM K^+ and dialyzed from the pipette with a buffer containing 140 mM K^+ and pH_{lum} 4.7. Zero current levels are indicated left of individual current responses by black bars. Corresponding current/voltage-relationships are shown below the current traces. **(B)** Averaged 4-AP-sensitive currents calculated from I/V-relationships as in (A) for ctrl (gray, n=5) and TMEM175-expressing lysosomes (blue, n=8). Data points represent arithmetic mean ± SEM. **(C)** Effect of 1 mM 4-AP on the current measured at +120 mV of n=5 ctrl and n=8 TMEM175-expressing lysosomes. Black lines connect values recorded from the same lysosome. Statistical comparisons were performed using a ratio paired t-test. **(D)** Representative current responses to voltage-step protocols from -120 mV to +120 mV in 20 mV increments of lysosomes (LY) from empty vector transfected (ctrl), TMEM175 wt- and TMEM175-H57Y expressing HEK293 cells recorded 3 min after achievement of whole-lysosome configuration. Lysosomes were bathed in a buffer with pH 7.4 and 140 mM K^+ and internally dialyzed from the pipette with a buffer containing 140 mM K^+ with either pH_{lum} 7.4 (upper row) or 4.7 (lower row). **(E)** Free running membrane potentials from whole-lysosome recordings as in (D). **(F,G)** Current densities at +120 mV **(F)** and -120 mV **(G)** calculated from current responses to voltage-step protocols as in (D). Bars in (E-G) represent arithmetic mean ± SD; values from individual recordings are shown as closed circles. Statistical comparisons were performed using a Brown-Forsythe ANOVA test with Dunnett's T3 multiple comparison test.

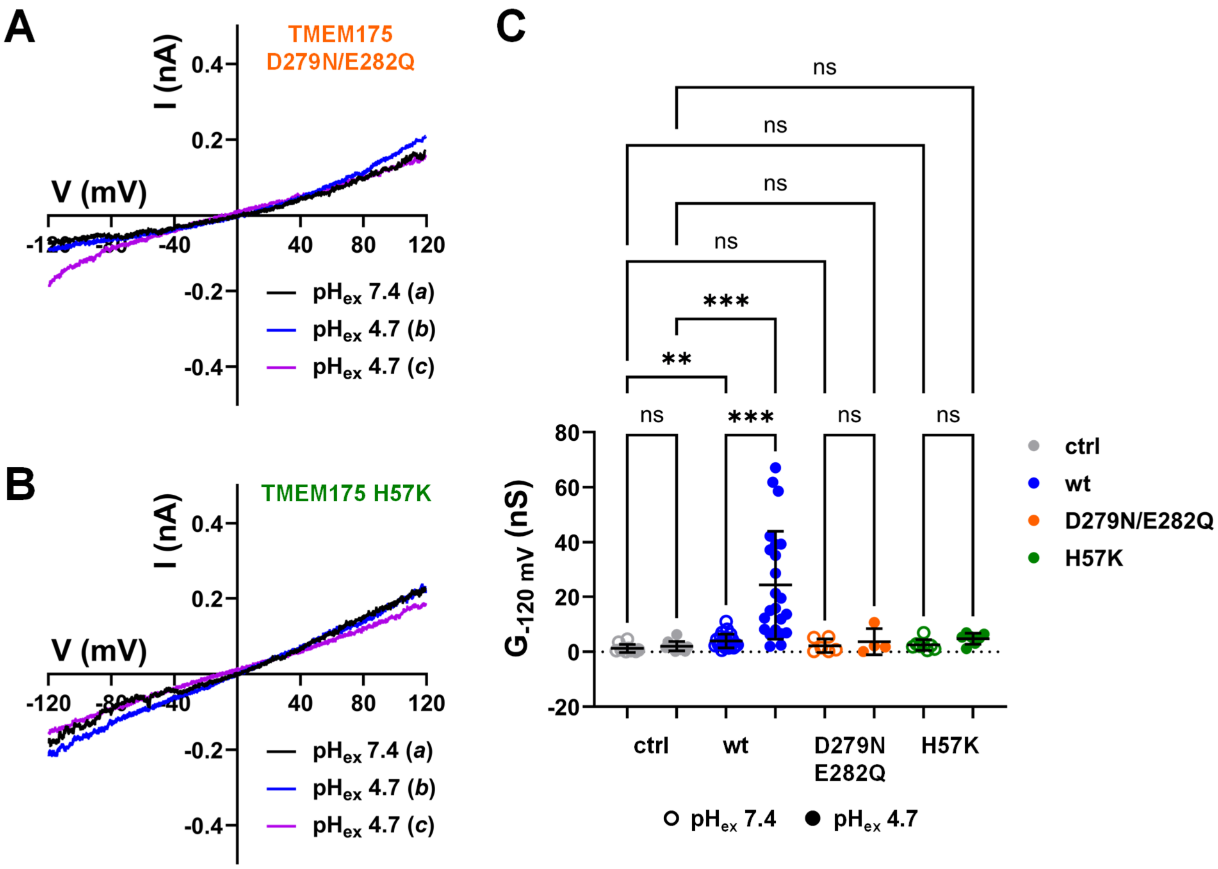

Fig.S10 Substitution of amino acids H57, D279, and E282 impairs the ion channel activity of TMEM175 in whole-cell patch-clamp recordings. (A,B) Representative current responses of TMEM175 D279N/E282Q- **(A)** and TMEM175 H57K- **(B)** expressing HEK293 cells to voltage-ramps from +120 mV to -120 mV shortly before (*a*, black), shortly after (*b*, pink) and 3 min after (*c*, purple) a pH_{ex}-jump from 7.4 to 4.7. The experiments were performed as in Fig.1. Indices *a*-*c* correspond to time points in Fig.1A$_{(ii)}$. **(C)** Chord conductance at -120 mV calculated from current responses as in (A) and (B) recorded shortly before (open circles) and 3 min after (closed circles) the pH_{ex}-jump from 7.4 to 4.7. Bars represent arithmetic mean ± SD. Statistical comparisons were performed using a Brown-Forsythe ANOVA test with Dunnett's T3 multiple comparison test.

Table S1 Software used for MD simulations, data analysis and visualization.

Software	Version	Source	Link
Biotite	1.0.1	(2)	https://www.biotite-python.org/latest/index.html
Gromacs	2021.5	(3)	https://www.gromacs.org/
Matplotlib	3.9.2	(4)	https://matplotlib.org/
NumPy	2.1.3	(5)	https://numpy.org/
Pandas	2.2.3	(6)	https://pandas.pydata.org/
Python	3.12.7	-	https://www.python.org/
Seaborn	0.13.2	(7)	https://seaborn.pydata.org/

Supplementary References

1. A. Bazzone, *et al.*, A Comparative Study on the Lysosomal Cation Channel TMEM175 Using Automated Whole-Cell Patch-Clamp, Lysosomal Patch-Clamp, and Solid Supported Membrane-Based Electrophysiology: Functional Characterization and High-Throughput Screening Assay Development. *Int J Mol Sci* **24**, 12788 (2023).
2. P. Kunzmann, *et al.*, Biotite: new tools for a versatile Python bioinformatics library. *BMC Bioinformatics* **24**, 236 (2023).
3. M. J. Abraham, *et al.*, GROMACS: High performance molecular simulations through multi-level parallelism from laptops to supercomputers. *SoftwareX* **1–2**, 19–25 (2015).
4. J. D. Hunter, Matplotlib: A 2D Graphics Environment. *Comput Sci Eng* **9**, 90–95 (2007).
5. C. R. Harris, *et al.*, Array programming with NumPy. *Nature* **585**, 357–362 (2020).
6. W. McKinney, Data Structures for Statistical Computing in Python in (2010), pp. 56–61.
7. M. Waskom, seaborn: statistical data visualization. *J Open Source Softw* **6**, 3021 (2021).
